# Lysine acetyltransferase NuA4 and acetyl-CoA regulate glucose-deprived stress granule formation in *Saccharomyces cerevisiae*

**DOI:** 10.1371/journal.pgen.1006626

**Published:** 2017-02-23

**Authors:** Meaghen Rollins, Sylvain Huard, Alan Morettin, Jennifer Takuski, Trang Thuy Pham, Morgan D. Fullerton, Jocelyn Côté, Kristin Baetz

**Affiliations:** 1 Ottawa Institute of Systems Biology, uOttawa, Ottawa, Ontario, Canada; 2 Department of Biochemistry, Microbiology and Immunology, uOttawa, Ottawa, Ontario, Canada; 3 Department of Cellular and Molecular Medicine, uOttawa, Ottawa, Ontario, Canada; University of Toronto, CANADA

## Abstract

Eukaryotic cells form stress granules under a variety of stresses, however the signaling pathways regulating their formation remain largely unknown. We have determined that the *Saccharomyces cerevisiae* lysine acetyltransferase complex NuA4 is required for stress granule formation upon glucose deprivation but not heat stress. Further, the Tip60 complex, the human homolog of the NuA4 complex, is required for stress granule formation in cancer cell lines. Surprisingly, the impact of NuA4 on glucose-deprived stress granule formation is partially mediated through regulation of acetyl-CoA levels, which are elevated in NuA4 mutants. While elevated acetyl-CoA levels suppress the formation of glucose-deprived stress granules, decreased acetyl-CoA levels enhance stress granule formation upon glucose deprivation. Further our work suggests that NuA4 regulates acetyl-CoA levels through the Acetyl-CoA carboxylase Acc1. Altogether this work establishes both NuA4 and the metabolite acetyl-CoA as critical signaling pathways regulating the formation of glucose-deprived stress granules.

## Author summary

In response to environmental stress, such as nutrient limitations or toxic chemicals, cells must quickly counteract these threats in order to survive. One way cells fight environmental challenges is through the formation of stress granules, which are aggregates of proteins and mRNA within the cytoplasm. Though their formation is essential for survival under multiple conditions, when stress granules are inappropriately formed they become causative for diseases such as amyotrophic lateral sclerosis and fragile X syndrome. Further stress granules contribute to chemotherapy resistance of cancer cells by promoting survival. Therefore it is critical to understand how stress granules are formed and disassembled. Here using the budding yeast *Saccharomyces cerevisiae* we determine that an enzyme called NuA4 is contributing to stress granule formation upon glucose deprivation. Why is this important? We determine that Tip60, the equivalent of NuA4 in mammalian cells, is also regulating stress granule formation in cancer cells. As there is a growing number of drugs that target this class of enzyme, there is the possibility that these drugs may reduce stress granule formation offering a novel therapeutic approach to treat numerous diseases.

## Introduction

When eukaryotic cells are exposed to environmental stress such as extreme temperatures, nutrient deprivation, or toxic chemicals, they quickly employ defense mechanisms to promote survival. An essential part of the stress response is the formation of stress granules (SGs), cytoplasmic ribonucleoprotein (RNP) granules that contain translationally repressed messenger RNA (mRNAs) and numerous other proteins. Not only is accumulation of SGs a hallmark of many neurodegenerative diseases, but mutations in known SG proteins that increase their tendency to aggregate are causative [reviewed in 1]. There is also growing evidence that SGs are contributing to both progression and chemotherapy resistance of cancer cells by promoting survival and inhibiting apoptosis [2–6]. The implication of stress granules as contributors to disease and chemotherapy resistance illustrates the importance of defining how this stress response is regulated within the cell.

Stress granule formation is a conserved cellular stress response and SGs are hypothesized to be triage centres where mRNAs are protected in a non-translating state prior to either being sorted to Processing Bodies (P-bodies) for degradation or back into active translation. Further mRNAs can also move from P-bodies to SGs, highlighting the dynamic nature of these RNP structures. Though P-bodies and SGs share some of the same protein components [7], SGs and P-bodies form independently in humans [8, 9] and under most stress conditions in yeast [10–12]. However, there is evidence that P-bodies promote formation of glucose deprivation SGs (GD-SGs) in yeast [13]. Although composition of SGs varies with stress, the core constituents of SGs are largely conserved and include poly(A)+mRNA, 40S ribosomal subunit, translation initiation factors, PolyA-binding protein Pab1, along with a number of proteins implicated in SG assembly such as Pub1/TIA-1, and Pbp1/ATAXIN-2 (yeast/MAMMALIAN names) [reviewed in 14]. These dynamic mRNPs also sequester proteins from key cell signaling pathways including pro-apoptosis factors and other signaling proteins which may protect the cell from initiating apoptosis during modest stress [15].

As SG-inducing stresses all cause inhibition of translation initiation and as drugs that trap mRNA in polysomes inhibit SG formation [13, 16, 17], it is generally believed that blocking translation initiation is necessary for SG assembly. However, not all stresses that inhibit translation initiation cause SG formation [13, 18], indicating that inhibition of translation is not the sole “trigger” of SG assembly but requires other signaling pathways, which like the composition of SGs are proposed to be stress-dependent. As SGs are transient structures that can assembly and dissolve within minutes even when translation initiation is inhibited, it is strongly argued that SG dynamics is regulated largely through post-translational modification (PTMs) [reviewed in 1].

In yeast, SGs have been documented to form under a variety of stress conditions, however GD-SG formation is by far the most characterized [13, 19, 20]. SGs form rapidly within 5 and 10 minutes after glucose deprivation and their formation are partially dependent on assembly factors Pbp1 and Pub1 [13]. Surprisingly the signaling pathway(s) triggering SG assembly upon glucose deprivation is poorly understood. Of the three primary glucose sensing and signaling pathways in *S*.*cerevisiae* two, TOR and PKA, do not appear to play a role in regulating GD-SG formation [21, 22]. It has been suggested that the third pathway, AMPK1/SNF1 may play a signaling role in glucose-deprived stress granule formation potentially through the activation of PAS kinases Psk1 [23]. However it remains to be tested if Snf1-activation of Psk1 is actually driving GD-SG formation.

Though the role of lysine acetylation in GD-SG dynamics in yeast has not been assessed, there is growing evidence that lysine acetylation is playing a critical role in various aspects of SG dynamics. In mammalian cells lysine acetyltransferase (KAT) CBP and lysine deacetylases (KDACs) HDAC6 and SIRT6 have been shown to both co-immunoprecipitate and co-localize with SG proteins [24–26]. In mouse embryonic fibroblasts (MEF) cells SIRT6 has been implicated in both the assembly and disassembly of heat-shock SGs [25], but the role of HDAC6 is not as straight forward. The downregulation of HDAC6 activity in MEF cells inhibited SG formation [26], while in QBI-293 cells it increased SG formation [24]. In the later study it was determined that acetylation of TDP-43 by CBP drove its aggregation into stress granules, while HDAC6-dependent deacetylation of TDP-43 reduced SG formation. In *S*. *cerevisiae* there are also intriguing hints that lysine acetylation contributes to SG dynamics. Deletion mutants of KAT *GCN5* and KDACs *HST1* and *SIR2* were identified to exhibit increased SG formation under non-stress conditions in a high content microscopy screen [10]. Furthermore we recently determined that Esa1, the catalytic domain of the yeast KAT NuA4, co-purified SG components Pab1, Pbp1, Lsm12 and Pbp4 and that both Pab1 and Pbp1 are *in vitro* targets of NuA4 [27].

Lysine acetyltransferases maybe exceptional signaling effectors for monitoring glucose and the metabolic state of the cell as their activity is intimately linked to acetyl-CoA, their acetyl donor [28–31]. Acetyl-coA is a central metabolite whose cellular levels are tightly linked to nutrient availability [reviewed in 32, 33, 34]. In *S*.*cerevisiae* there are two pools of acetyl-CoA, one mitochondrial and one nucleocytosolic, the latter of which affects nuclear and cytosol lysine acetylation events. Under conditions where glucose is in abundance, nucleocytosolic levels of acetyl-CoA and histone acetylation are high. However once cells exhaust their sources of glucose, acetyl-CoA and histone acetylation decrease [28, 35, 36]. Even within a cell cycle, oscillations of acetyl-CoA levels have been shown to regulate KAT activity against both histones and other substrates [28]. Further studies in both yeast and human cells show that nucelocytosolic depletion of acetyl-CoA induces autophagy, largely through KAT-dependent transcriptional regulation of autophagy genes [37, 38].

Given that KATs are naturally designed to monitor nutrient availability and the clear links between lysine acetylation and SG dynamics, we sought to determine if KATs contribute to the regulation of GD-SG formation in yeast. We demonstrate that NuA4 is required for the assembly of glucose-deprived stress granules and identify a conserved role for Tip60, the mammalian homolog of Esa1 [39], in the regulation of SGs in human breast cancer cells. Surprisingly, we find that NuA4 is partially regulating the formation of GD-SGs through acetyl-CoA levels. Together our work defines novel signaling pathways that regulates SG dynamics upon glucose deprivation and suggests that NuA4 regulation of acetyl-CoA levels is likely mediated through Acc1.

## Results

### NuA4 is required for glucose-deprived stress granule assembly but not processing body assembly

**Fig 1:**
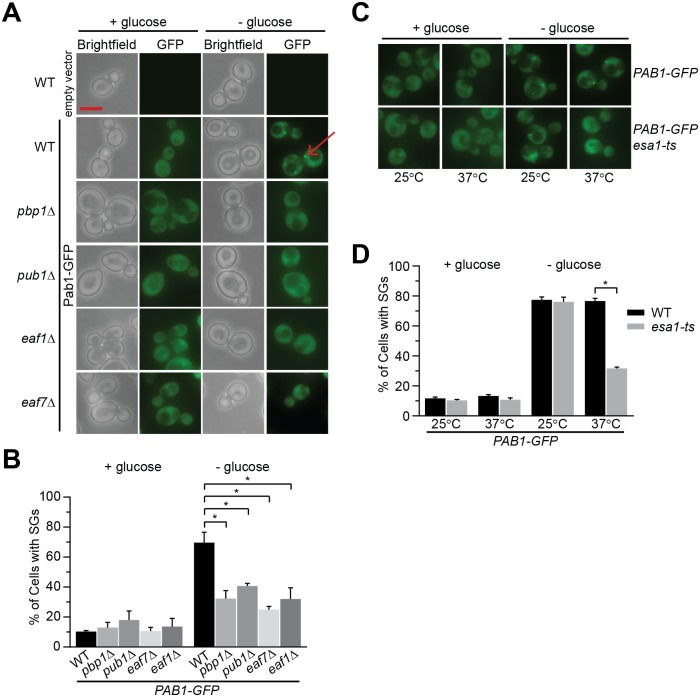
NuA4 mutant cells exhibit reduced Pab1-GFP cytoplasmic foci upon glucose deprivation. **A&B**) Wild type (WT, YKB3263), *pbp1Δ* (YKB3262), *pub1Δ* (YKB3261), *eaf7Δ* (YKB3729), and *eaf1Δ* (YKB3260) transformed with *PAB1-GFP*::*URA*::*CEN* plasmid (pBK192) and WT transformed with empty vector were cultured in SCD-URA medium (+glucose) at 30°C and exponential-phase cells were subjected to 10 minutes of GD (-glucose) and immediately accessed for Pab1-GFP foci (SGs). **A**) Representative brightfield and florescent images. **B**) Quantification of the percentage of cells displaying at least one SG. **C & D**) WT (YKB3114) and *esa1-ts* (YKB3855) cells expressing endogenously tagged Pab1-GFP were cultured in YPD medium at 25°C to mid-exponential phase. Half the culture was subjected to 10 minutes of pre-warmed 37°C YPD medium prior to 10 minutes of GD and immediately assessed for SGs. **C**) Representative florescent images. **D**) Quantification of the percentage of cells with SGs. Arrow highlights Pab1-GFP foci; red scale bar: 5 μm. Results are the average of the three biological replicates, a minimum of 100 cells per replicate were scored, error bars indicate the standard error of the mean (SEM). * denotes statistical significance at a p-Value < 0.05 determined using a two-way ANOVA test.

**Fig 2:**
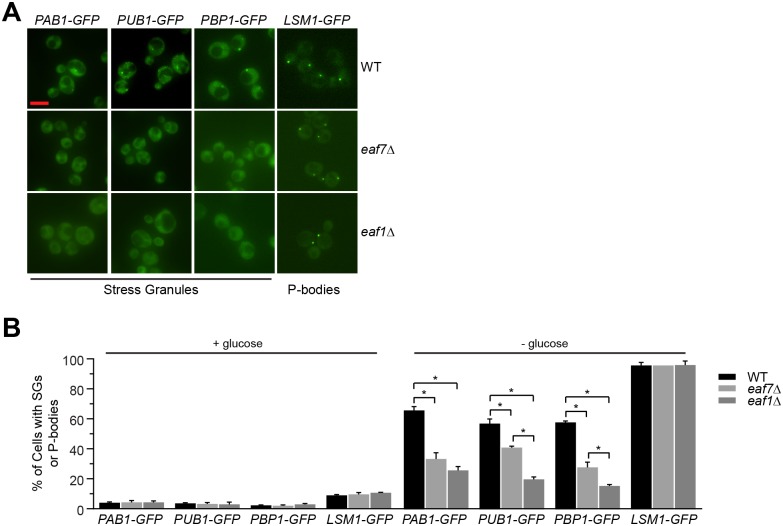
NuA4 is required for glucose deprivation stress granule formation but does not impact processing bodies. Exponential-phase cells expressing endogenously tagged Pab1-GFP (WT YKB3114; *eaf7*Δ YKB3336; *eaf1*Δ YKB3382), Pub1-GFP (WT YKB3115; *eaf7*Δ YKB3337; *eaf1*Δ YKB3339), Pbp1-GFP (WT YKB3258; *eaf7*Δ YKB3335; *eaf1*Δ YKB3338) or Lsm1-GFP (WT YKB3710; *eaf7*Δ YKB3718; *eaf1*Δ YKB3717) cultured YPD medium at 30°C were subjected to 10 minutes of GD and immediately scored for SGs. **A**) Representative florescent images. Scale bar: 5 μm. **B**) Quantification of percentage of cells with SGs or P-body foci. Results are the average of the three biological replicates, a minimum of 100 cells per replicate were scored, error bars indicate the standard error of the mean (SEM). Error bars indicate the SEM. * denotes statistical significance at a p-Value < 0.05 determined using a two-way ANOVA test.

Given the interaction between Esa1 and stress granule proteins [27] and the roles of NuA4 in glucose metabolism [40, 41], we sought to determine whether NuA4 has a role in SG formation upon glucose deprivation. NuA4 is a multi-subunit complex, composed of the essential catalytic subunit Esa1 [42], five other essential subunits Act1, Arp4, Epl1, Swc4, Tra1, and seven non-essential subunits Eaf1, Eaf3, Eaf5, Eaf6, Eaf7, Yaf9, and Yng2 [39]. Molecular and structural dissection has revealed NuA4 to be modular in nature [43], and that assembly of its multiple sub-complexes depends on the Eaf1 subunit [44, 45]. Wild type, mutants of core SG subunits *pbp1Δ* and *pub1Δ*, along with NuA4 non-essential mutants *eaf1Δ* and *eaf7Δ* were transformed with a Pab1-GFP expressing plasmid [46]. *pbp1*Δ and *pub1*Δ cells were used as controls as both mutants display reductions in GD-SGs [13]. The percentage of cells with Pab1-GFP foci and the number of foci/cell (S1 Table contains all quantification for strains studied) were quantified in both 2% glucose media and after 10 minutes of glucose deprivation. None of the mutants examined display a significant increase or constitutive formation of SGs under glucose conditions compared to wild type. As expected, upon glucose deprivation Pab1-GFP SGs are induced in wild type cells but the induction was significantly reduced in *pub1Δ* and *pbp1Δ* cells [13] (Fig 1A and 1B). Upon glucose deprivation *eaf1Δ* and *eaf7Δ* cells show a decrease in Pab1-GFP SGs to a similar extent as *pbp*1Δ and *pub1*Δ cells (Fig 1A and 1B). Decreases in GD-SG formation were seen for NuA4 mutants’ *eaf3Δ* and *eaf5Δ* (S2 Table). Furthermore, while the temperature-sensitive allele of *ESA1*, *esa1-L254P* (*esa1-ts*) [42], form GD-SGs at the permissive temperature (25°C), when pre-incubated at the non-permissive temperature (37°C) prior to 10 minutes of glucose deprivation, there is a significant decrease in Pab1-GFP foci formation (Fig 1C and 1D), indicating that the catalytic activity of NuA4 is required for Pab1-GFP SG formation. To determine if NuA4 is only promoting Pab1 assembly into GD-SGs or whether it had a greater role in SG or P-body formation we examine the localization of endogenously tagged core stress granule proteins Pbp1-GFP, Pub1-GFP, and Pab1-GFP [13, 20] along with the P-body marker Lsm1-GFP [47] in wild type, *eaf1*Δ and *eaf7*Δ strains after 10 minutes of glucose deprivation. Similar to plasmid based Pab1-GFP experiment (Fig 1), NuA4 mutants display a decrease in endogenously tagged Pab1-GFP localization to GD-SGs (Fig 2A & 2B). In addition similar defects were observed for Pub1-GFP and Pbp1-GFP, indicating that NuA4 is impacting GD-SG assembly, and not just Pab1-GFP localization to GD-SGs. Though decreased levels of Pab1-GFP and Pub1-GFP were detected in *eaf1Δ* cells, deletion of *EAF7* had no effect on protein levels (S1 Fig), suggesting that defects in glucose-deprived SG formation is not due to changes of the SG marker levels. In contrast, NuA4 mutants did not impact P-body marker Lsm1-GFP foci formation. While NuA4 is required for glucose-deprived SG formation it is not required for the formation of heat-shock or ethanol SGs (S2 Fig). Taken together, this work has identified NuA4 as a novel signaling pathway for formation of SGs upon glucose deprivation.

### Functionally redundant roles for Eaf7 and Gcn5 in SG formation upon glucose deprivation

**Fig 3:**
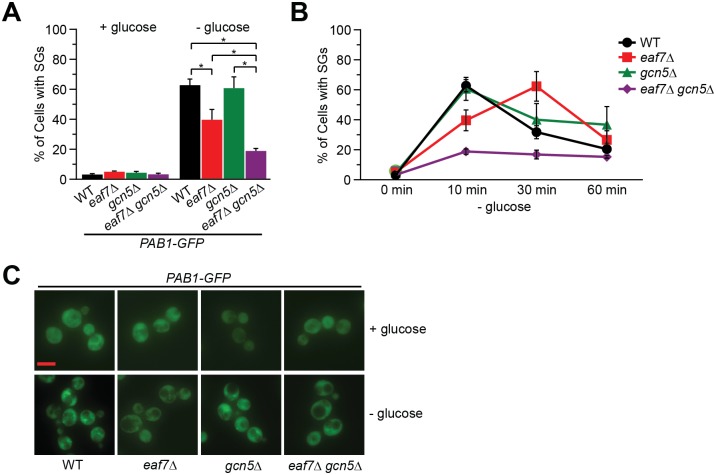
Gcn5 can partially compensate for the absence of Eaf7 in formation of glucose-deprived stress granules. WT (YKB3114), *eaf7*Δ (YKB3336), *gcn5*Δ (YKB4116), and *eaf7*Δ*gcn5*Δ (YKB4118), cells expressing endogenously tagged Pab1-GFP were grown to mid-log phase at 30°C in YPD medium (0 minutes) were subjected to GD (-glucose) for 10, 30 and 60 minutes and immediately assessed for SGs. **A**) Quantification of the percentage of cells with SGs after 10 minutes GD. **B**) Representative florescent images. Scale bar: 5 μm. **C**) Time course of the percentage of cells with SGs. Results are the average of the three biological replicates, a minimum of 100 cells per replicate were scored, error bars indicate the SEM. * denotes statistical significance at a p-Value < 0.05 determined using a two-way ANOVA test.

To determine if other KATs or KDACs in *S*. *cerevisiae* play a role in GD-SG formation we systematically screened a library of single non-essential KAT and KDAC mutants (S2 Table). The KAT/KDAC mutant library was transformed with a Pab1-GFP expressing plasmid and screened for SGs under both glucose replete and depleted conditions. In addition to NuA4 mutants (*eaf1Δ*, *eaf3Δ*, *eaf5Δ* and *eaf7Δ*), deletion mutants of the KAT *GCN5* also displayed a modest decrease in GD-SG formation. Therefore, we sought to determine if Gcn5 does impact GD-SGs and/or if it has a functionally redundant role with NuA4 in GD-SG dynamics. Glucose-deprived stress granule formation was measured in wild type, *eaf7Δ*, *gcn5Δ*, and *eaf7Δgcn5Δ* cells expressing Pab1-GFP from its endogenous loci in both glucose and glucose deprivation conditions (10 minutes, 30 minutes and 60 minutes). *eaf7Δ* was selected as a proxy for NuA4 as it displays defects in GD-SG, but unlike *eaf1Δ*, *eaf7*Δ cells displays minimal fitness defects [48]. Furthermore, while *eaf1*Δ*gcn5Δ* cells are inviable [45, 49, 50], *eaf7Δgcn5Δ* cells display only minor growth defects (S2C Fig). While *gcn5*Δ cells did not display a significant reduction in GD-SG assembly compared to wild type cells using the endogenously integrated Pab1-GFP, *eaf7Δgcn5Δ* cells display a significant reduction in GD-SG formation at 10 minutes compared to both wild type and single KAT mutants (Fig 3A and 3B). To better understand the contribution of Eaf7 and Gcn5 to kinetics of SG formation/disassembly, SGs were measured over 10, 30 and 60 minutes glucose deprivation (Fig 3C). As previously shown in wild type cells GD-SGs quickly form and then start resolving by 30 minutes as the cells begin to adapt to glucose deprivation [11]. While *gcn5*Δ cells had no impact on GD-SGs over the time course, *eaf7*Δ cells display a delay in the formation of GD-SGs. In contrast, the percentage of cells with SGs do not increase over the glucose deprivation time course in *eaf7*Δ*gcn5*Δ cells. As *eaf7Δgcn5Δ* cells display minimal growth defects compared to the single mutants, it is unlikely the defects in GD-SG formation reflect cell sickness. Rather these results suggest that *EAF7* and *GCN5* are functionally redundant in GD-SG regulation and in the absence of Eaf7, and presumably NuA4, Gcn5 is able to compensate albeit with slower kinetics for GD-SG formation.

### Tip60 affects stress granule levels in mammalian cells

**Fig 4:**
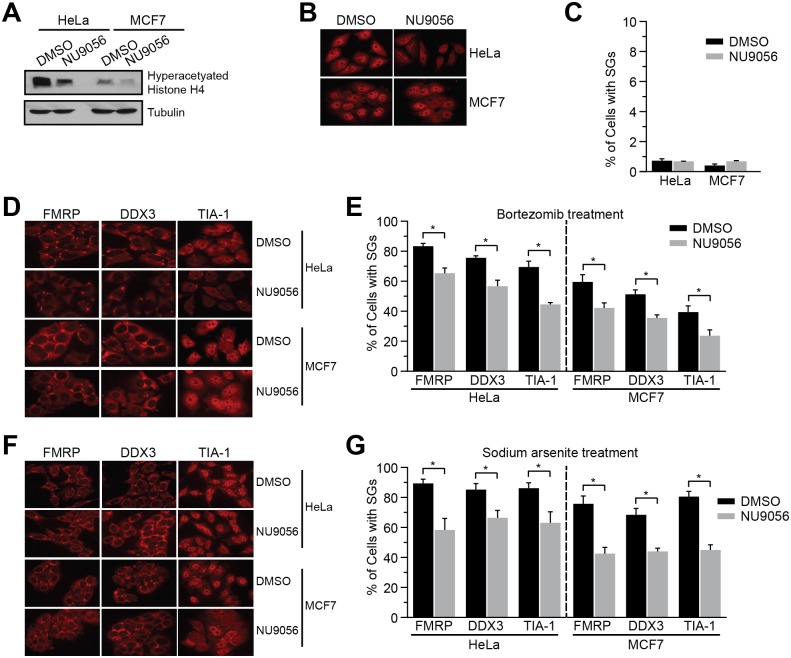
TIP60 inhibition reduces stress granule formation in mammalian cells. **A**) Decreased acetylation level of histone H4 was detected by Western Blot analysis upon treatment with NU9056. HeLa and MCF7 cells were treated with DMSO or 20 μM NU9056 for 24 hours, and histone extraction and western blot analysis was performed with the indicated antibodies. **B**) NU9056 does not promote SG formation in HeLa and MCF7 cells. HeLa and MCF7 cells were treated with DMSO or 20 μM NU9056 for 24 hours and immunofluorescences was performed using TIA-1 as marker for SG formation. **C**) Quantification the average percentage of cells with NU9056-induced SGs. **D**) Treatment of HeLa and MCF7 cells with NU9056 resulted in a significant decrease of bortezomib-induced SG formation. HeLa and MCF7 cells were treated with DMSO or 20 μM NU9056 for 24 hours, and then 10 μM bortezomib for 4 hours. Immunofluorescence was performed using FMRP, DDX3, and TIA-1 as markers of SG formation. **E**) Quantification the average percentage of cells with bortezomib-induced SGs. **F**) Treatment of HeLa and MCF7 cells with NU9056 resulted in a significant decrease of sodium arsenite-induced SG formation. HeLa and MCF7 cells were treated with DMSO or 20 μM NU9056 for 24 hours, and then 100 μM sodium arsenite for 1 hour. Immunofluorescence was performed using FMRP, DDX3, and TIA-1 as markers of SG formation. **G**) Quantification of the average percentage of cells with sodium-arsenite induced SGs. Results are the average of three independent experiments with at least 500 cells counted per stress granule marker in each independent experiment. Error bar represent the standard error of the mean. * denotes statistical significance at a p-Value < 0.05 determined using an unpaired t-test.

Given the conservation of KAT complexes and stress granule biology, we predicted that the Tip60 complex, the mammalian homolog of the NuA4 complex, might also contribute to SG dynamics. To test this possibility we examined SGs in HeLa and MCF7 cell lines in the presence and absence of the inhibitor NU9056 which inhibits the activity of the Tip60 protein, the catalytic subunit of the complex and the mammalian homolog of *S*. *cereviaise* Esa1 [51]. While cells treated with 20 μM NU9056 alone for 24 hours resulted in a decrease in acetylated histone H4 (Fig 4A) it did not promote SG formation in unstressed mammalian cells as assessed using the SG marker TIA-1 [17] (Fig 4B and 4C). We next asked whether inhibition of Tip60 decreases SG formation promoted by sodium arsenite or bortezomib, a chemotherapeutic drug that has been shown to induce SG formation in bortezomib-resistant cancer cell lines [4]. HeLa and MCF7 cells were treated with NU9056 for 24 hours and then either 10 μM bortezomib for 4 hours or 100 μM sodium arsenite for 1 hour. Stress granule formation was analyzed by immunofluorescence using the SG markers TIA-1, FMRP [52], and DDX3 [53]. Treatment with NU9056 resulted in a significant decrease in bortezomib-induced (Fig 4D & 4E) and sodium arsenite-induced (Fig 4F & 4G) SGs in both cell types. These results show that NuA4/Tip60 have a conserved function in regulating SG dynamics.

### NuA4 regulation of glucose deprivation stress granules is not mediated through inhibition of translation initiation or Snf1

**Fig 5:**
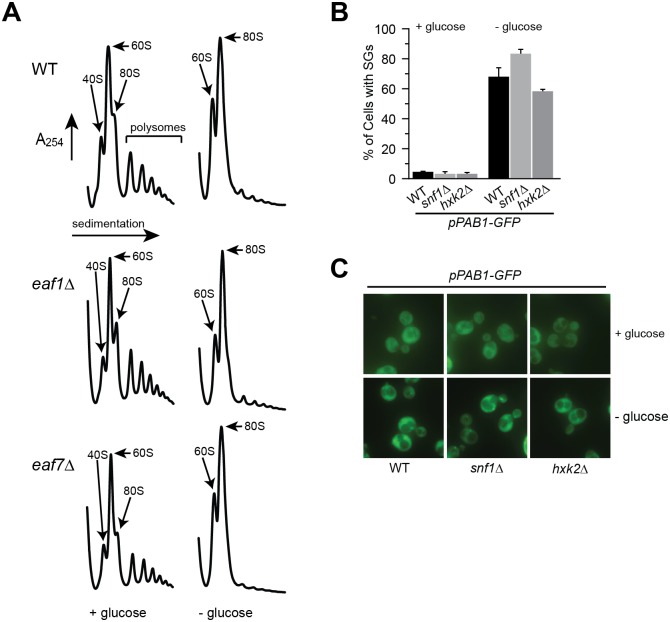
NuA4 does not regulate the formation of stress granules through the inhibition of translation initiation or the Snf1 pathway. **A**) Eaf1 and Eaf7 are not required for the inhibition of translation initiation upon 10 minutes glucose deprivation. Polyribosome profiles of WT (YKB1079), *eaf1*Δ (YKB3333) and *eaf7*Δ(YKB3292) cells that were grown to mid-log phase at 30°C in YPD (+glucose) and then subjected to glucose deprivation for 10 minutes (-glucose). The peaks that contain the small ribosomal subunit (40S), the large ribosomal subunit (60S), and both subunits (80S) are indicated by arrows. The polysome peaks are bracketed. **B**) *SNF1* is not required for the glucose-deprived SG assembly. WT (YKB3114), *snf1*Δ (YKB4342) and *hxk2*Δ (YKB4343) cells expressing endogenously tagged Pab1-GFP were grown to mid-log phase at 30°C in YPD medium (+glucose), subjected to glucose deprivation for 10 minutes (-glucose) and assessed for SGs. Quantification of percentage of cells with SGs. Results are the average of the three biological replicates, a minimum of 100 cells per replicate were scored, error bars indicate the SEM. * denotes statistical significance at a p-Value < 0.05 determined using unpaired t-test. **C**) Representative florescent images.

We next sought to determine if the signaling pathway through which NuA4 regulates glucose-deprived SG formation was through either regulation of translation initiation or SNF1/AMPK pathway. Though NuA4 has not been implicated in translation initiation, it does have a role in the transcriptional regulation of ribosome genes [54] and both genetic and physical interactions with ribosome subunits [27, 45, 49]. If NuA4 mutant defects in GD-SG formation were due defects in inhibition of translation upon glucose deprivation we would anticipate that in NuA4 mutants polysomes would remain associated with RNA upon glucose deprivation. However, this is not the case as ribosomal profiling determined that like wild type cells, *eaf1Δ* and *eaf7Δ* cells are capable of stalling translation initiation by polysome disassembly in response to glucose deprivation (Fig 5A). Another candidate pathway through which NuA4 might regulate GD-SG formation is the SNF1/AMPK pathway, a major sensor of glucose state that is regulated by NuA4 acetylation of Sip2 [41]. Sip2 is one of three β-regulatory subunits of the Snf1 complex and a repressor of Snf1/AMPK activity. NuA4 acetylation of Sip2 enhances its interaction and inhibition of Snf1. In contrast, in NuA4 mutants Sip2 acetylation decreases, destabilizing its interaction with Snf1, leading to increased Snf1 activity. If NuA4 mutant defects in GD-SG formation were due to hyperactive Snf1, we would anticipate that other mutants with hyperactive Snf1 would display defects in SG-formation. However, *hxk2*Δ cells which have hyperactive Snf1 [55] do not have defects in GD-SG formation (Fig 5B and 5C). Surprisingly, we also determined that in cells in which *SNF1* is deleted, GD-SGs still occurs (Fig 5B and 5C) indicating that Snf1 kinase activity is not required for the assembly of Pab1-GFP into glucose-deprived SGs. Together this shows that NuA4 is contributing to GD-SG dynamics through a novel mechanism.

### Glucose deprivation stress granules are suppressed by exogenous acetate

**Fig 6:**
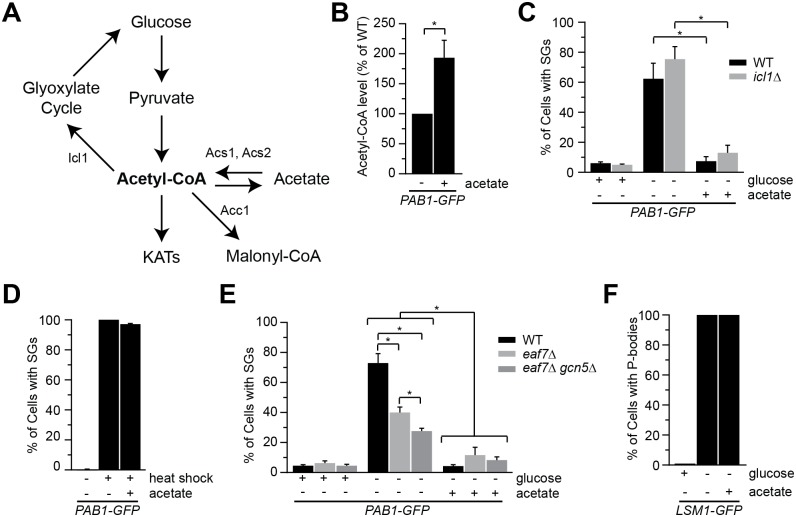
Exogenous acetate suppresses glucose-deprived stress granule formation. **A**) Schematic illustrating acetyl-CoA biosynthesis pathways. **B**) Acetate treatment of cells increase cellular acetyl-CoA levels. WT cells expressing endogenously tagged Pab1-GFP (YKB3114) were grown to mid-log phase at 30°C in YPD medium, treated with 100 mM acetate for 10 minutes, and acetyl-CoA extraction and measurements were performed. Quantification of acetyl-CoA levels with acetate-treated cells normalized to untreated cells for three independent experiments, error bar indicates the SEM. * denotes statistical significance at a p-Value < 0.05 determined using an unpaired t-test. **C**) Acetate treatment suppresses GD-SG formation. WT (YKB3114) and *icl1*Δ (YKB4010) cells expressing endogenously tagged Pab1-GFP were grown to mid-log phase at 30°C in YPD medium, and subjected to either glucose deprivation for 10 minutes or glucose deprivation plus 100mM acetate for 10 minutes. **D**) Acetate treatment does not suppress heat-shock induced SGs. WT cells expressing endogenously tagged Pab1-GFP (YKB3114) were grown to mid-log phase at 30°C in YPD, subjected to heat-shock at 46°C for 10 minutes with or without 100mM acetate treatment. Quantification of the percentage of cells with SGs. **E)** Acetate treatment does not impact P-body formation. WT cells expressing Lsm12-GFP (YKB3710) were grown to mid-log phase at 30°C in YPD medium, and subjected to either glucose deprivation for 10 minutes or glucose deprivation plus 100mM acetate for 10 minutes. **F**) Acetate treatment can further suppress GD-SG formation of *eaf7*Δ and *eaf7*Δ*gcn5*Δ cells. WT (YKB3114), *eaf7Δ* (YKB3336) and *eaf7Δgcn5Δ* (YKB4118) cells expressing endogenously tagged Pab1-GFP grown to mid-log phase at 30°C in YPD medium were subjected to glucose deprivation (-glucose) with or without 100 mM acetate treatment for 10 minutes. SG results are the average of the three biological replicates, a minimum of 100 cells per replicate were scored, error bars indicate the SEM. * denotes statistical significance at a p-Value < 0.05 determined using a two-way ANOVA test.

As cellular levels of acetyl-CoA reflect glucose availability, we next sought to determine if changes in acetyl-CoA impact glucose-deprived SG formation. As exogenous acetate treatment of *S*. *cerevisiae* is converted to acetyl-CoA (Fig 6A), we asked if glucose-deprived SG formation in wild type cells was impacted by exogenous acetate. As previously shown [28], treatment of exponential-phase wild type cells with 100 mM acetate results in increased acetyl-CoA levels (Fig 6B). We found that acetate treatment significantly reduced the number of cells with GD-SGs (Fig 6C and S3A Fig). As the repression by acetate might reflect conversion of acetate to glucose by the glyoxylate cycle we asked if acetate could still suppress GD-SGs in *icl1Δ* cells, a mutant that disrupts the glyoxylate cycle pathway [56] (Fig 6C and S3A Fig). Acetate could suppress GD-SGs in *icl1*Δ cells, indicating the suppression is not due to conversion of acetate to glucose but rather through another metabolite or mechanism. Further, galactose and metabolic intermediates citrate and pyruvate cannot suppress GD-SG formation suggesting the effect of acetate is not simply due to reintroduction of an energy source (S4A and S4B Fig). Interestingly 2% ethanol can partially suppress GD-SGs (S4A and S4B Fig) which might reflect conversion of ethanol to acetyl-CoA [57], though higher levels of ethanol induce SGs [58]. Similar to NuA4 mutants, acetate treatment did not suppress heat-shock (Fig 6D and S3B Fig) or ethanol SGs (S2D and S2E Fig) and acetate treatment did not impact P-body (Lsm1-GFP foci) formation (Fig 6E and S3C Fig). As increased acetyl-CoA results in increased protein acetylation [29, 30], we asked whether acetate suppression of GD-SGs requires Eaf7/NuA4 or Gcn5. We found that acetate treatment could further reduce the number of cells with GD-SGs in both *eaf7Δ* and *eaf7Δgcn5Δ* (Fig 6F and S3D Fig). Together this work indicates that exogenous acetate treatment can suppress GD-SG formation and this can occur through a mechanism independent of Eaf7/NuA4 or Gcn5 activity.

### Reduced expression of *ACC1* decreases glucose-deprived stress granule formation

**Fig 7:**
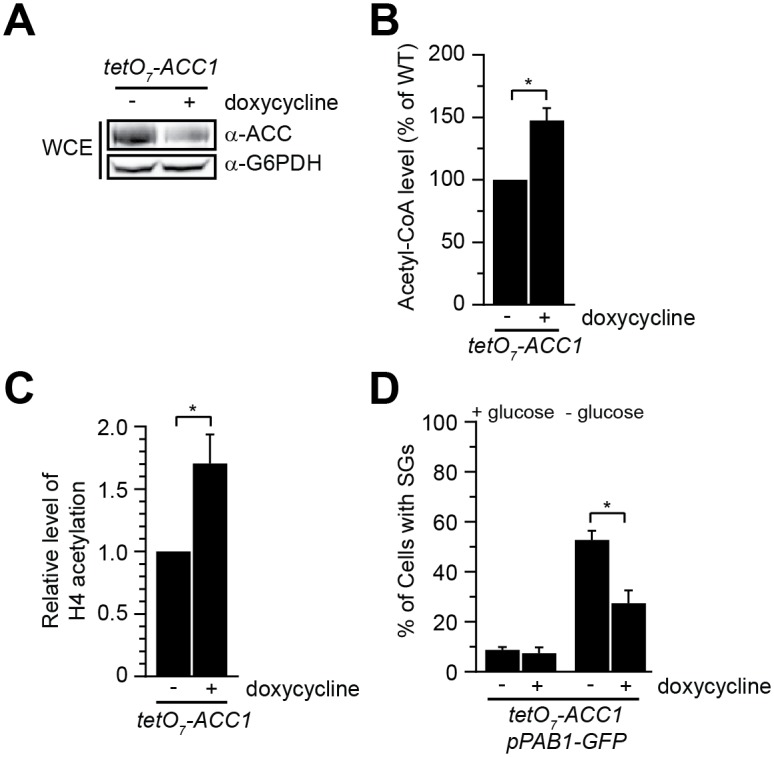
Reduction of Acc1 protein level decreases glucose-deprived stress granule formation. **A-C**) Reduction of Acc1 protein levels results in increased acetyl-CoA levels and histone H4 acetylation. tet07-*ACC1* (YKB4047) cells were grown to mid-log phase at 30°C in YPD medium, culture was split with one half being treated with 10 μM doxycycline for 2.5 hours. **A**) TCA protein extraction was performed and whole cell extracts (WCE) was resolved by SDS-PAGE prior to Western Blot analysis using the indicated antibodies. The image is representative of three replicates. **B**) Quantification of acetyl-CoA levels of three independent experiments. Acetyl-CoA levels are normalized to untreated cells, error bar represents SEM. **C**) Quantification of histone H4 acetylation of three independent experiments. 150 μg of WCE was resolved by SDS-PAGE prior to Western Blot analysis and quantification. Histone H4 acetylation level of doxycycline-treated cells are normalized to untreated cells, error bar represents the SEM. **D**) Reduction of Acc1 protein level prior to glucose deprivation suppresses SG formation. tet07-*ACC1* transformed with *PAB1-GFP*::*URA*::*CEN* plasmid (pBK192) were grown to mid-log phase at 30°C in SCD medium (+glucose). Half of the culture was treated with 10 μM doxycycline for 2.5 hours prior to glucose deprivation for 10 minutes. Cells were scored for SGs. Quantification of the percentage of cells with SGs. Results are the average of the three biological replicates, a minimum of 100 cells per replicate were scored, error bars indicate the SEM. * denotes statistical significance at a p-Value < 0.05 determined using a unpaired t-test (panel B&C) or two-way ANOVA test (panel D).

To confirm that the impact of exogenous acetate on SG formation is through the metabolite acetyl-CoA, we next sought to determine if genetic manipulation of cellular acetyl-CoA levels could impact GD-SG formation. Reducing the activity of the essential acetyl-CoA carboxylase Acc1 results in an increase in cellular acetyl-CoA [29]. A doxycycline regulated tet0_7_ repressible promoter was used to control the expression of the essential protein *ACC1* (*tet0*_*7*_*-ACC1*) [59]. Exponentially grown *tet0*_*7*_*-ACC1* cells transformed with a Pab1-GFP expressing plasmid were treated with either vehicle control or 10uM doxycycline for a 2.5 hour incubation period prior to glucose deprivation. As previously shown, doxycycline treatment results in reduced Acc1 protein levels, increased cellular acetyl-CoA levels, and increased histone acetylation (Fig 7A–7C). While, doxycycline treatment did not induce SGs in glucose replete conditions, reducing Acc1 activity suppressed glucose-deprived SG formation (Fig 7D and S5A Fig). Thus, reducing the expression of *ACC1*, correlates with increasing acetyl-CoA levels and a reduction in glucose-deprived SG formation.

### Suppression of glucose-deprived stress granule formation by *eaf7Δ* mutants is mediated by increased acetyl-CoA

**Fig 8:**
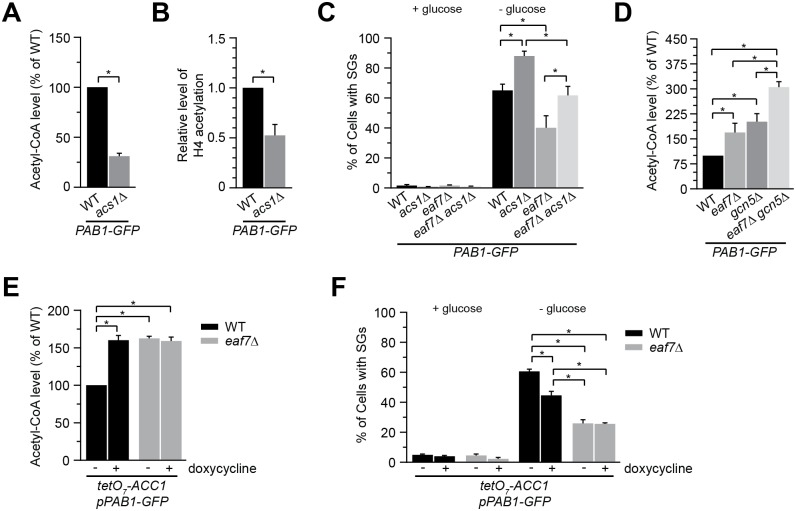
Eaf7 regulates glucose-deprived stress granule formation through both acetyl-CoA dependent and independent pathways. **A-C**) Deletion of *ACS1* increases the number of cells with glucose-deprived SGs. Exponential-phase WT (YKB3114) and *acs1*Δ (YKB4132) cells expressing endogenously tagged Pab1-GFP grown at 30°C in YPD medium were analyzed for acetyl-CoA or acetylation of Histone H4. **A**) Quantification of acetyl-CoA levels of three independent experiments. Acetyl-CoA levels are normalized to WT cells, error bar represents SEM. **B**) Quantification of histone H4 acetylation of three independent experiments. 150 μg of WCE was resolved by SDS-PAGE prior to Western Blot analysis and quantification. Histone H4 acetylation are normalized to untreated cells, error bar represents the SEM. **C**) Increased number of cells with GD-SGs in *acs1*Δ can be buffered by *eaf7*Δ. Exponential-phase WT (YKB3114), *acs1*Δ (YKB4132), *eaf7*Δ (YKB3336) and *acs1*Δ*eaf7*Δ (YKB4287) cells expressing endogenously tagged Pab1-GFP grown at 30°C in YPD (+glucose) were subjected to glucose deprivation (-glucose) for 10 minutes and immediately scored for SGs. Quantification of the percentage of cells with SGs. **D**) *eaf7Δ* and *gcn5Δ* mutant cells have elevated acetyl-CoA levels. Exponential-phase WT (YKB3114), *eaf7*Δ (YKB3336), *gcn5*Δ (YKB4116) and *eaf7*Δ*gcn5*Δ (YKB4118) cells expressing endogenously tagged Pab1-GFP grown at 30°C in YPD medium were analyzed for acetyl-CoA levels. Quantification of acetyl-CoA level of three independent experiments normalized to WT cells, error bar represents SEM. **E & F**) Eaf7 is epistatic to Acc1 and regulates glucose-deprived SG formation through both has acetyl-CoA dependent and independent pathways. tet07-*ACC1* (YKB4048) and tet07-*ACC1eaf7*Δ (YKB4249) transformed with *PAB1-GFP*::*URA*::*CEN* plasmid (pBK192) were grown to mid-log phase at 30°C in SCD medium and half the cultures were treated with 10 μM doxycycline for 2.5 hours prior to GD for 10 minutes and immediately assessed for (**E**) acetyl-CoA levels and (**F**) SGs (Pab1-GFP foci). **E**) *eaf7*Δ and *acc1* mutants do display additive increases in acetyl-CoA levels. Quantification of acetyl-CoA levels of three independent experiments. Acetyl-CoA levels are normalized to WT cells, error bar represents SEM. **F**) Eaf7 is epistatic to Acc1 and regulates glucose-deprived SG formation through both has acetyl-CoA dependent and independent pathways. Quantification of percentage of cells with SGs. Results for SG analysis are the average of the three biological replicates, a minimum of 100 cells per replicate were scored, error bars indicate the SEM. * denotes statistical significance at a p-value < 0.05 determined using a unpaired t-test (panels A,B,) or two-way ANOVA test (panels C,D,E,F).

If acetyl-CoA is regulating glucose-deprived SG formation, we predicted that decreasing cellular acetyl-CoA would increase SG formation. *Saccharomyces cerevisiae* have two acetyl-CoA synthetases, Acs1 and Acs2, which produce cellular acetyl-CoA from acetate. While *ASC2* is essential under glucose culture, the non-essential *ACS1* is induced upon glucose depletion and is required for survival on non-fermentable carbon sources [30, 60–62]. While we could not detect changes in GD-SG formation in either a strain carrying a temperature sensitive allele of *ACS2* [30] at 33°C or *tet0*_*7*_*-ACS2* strain treated with 10uM doxycycline (S6 Fig), this was not the case with *ACS1*. Exponentially grown *acs1Δ* cells have a significant decrease in acetyl-CoA levels and a decrease in histone H4 acetylation as compared to wild type cells (Fig 8A and 8B). Deletion of *ACS1* results in a significant increase in glucose-deprived SG formation (Fig 8C and S5B Fig). Interestingly, the increase in GD-SGs in *acs1*Δ cells was reduced to wild type GD-SG levels by deletion of *EAF7*. As the *acs1Δeaf7Δ* cells did not display phenotypes similar to *eaf7Δ* cells, it suggests that Acs1 and Eaf7 may contribute to SG dynamics through distinct pathways. Alternatively, deletion of *EAF7* may result in an increase in acetyl-CoA levels that could compensate for the reduction of acetyl-CoA seen in the absence of Acs1. To explore this hypothesis we measured acetyl-CoA levels in exponentially grown *eaf7Δ* and *gcn5Δ* cells and found that both have a significant increase in acetyl-CoA levels compared to wild type cells (Fig 8D). In addition, acetyl-CoA level are further increased in the *eaf7Δgcn5Δ* mutant, which parallels the increased suppression of GD-SGs seen in this mutant (Fig 3B). We next asked if the impact of Eaf7 on acetyl-CoA levels is in parallel or epistatic to Acc1 (Fig 8E). As previously shown *tet0*_*7*_*-ACC1* cells treated with doxycycline display an increase in acetyl-CoA, however acetyl-CoA levels do not significantly increase upon the deletion of *EAF7* (compare *eaf7*Δ*tet0*_*7*_*-ACC1* vs *tet0*_*7*_*-ACC1* treated with doxycycline). This suggests that Eaf7 and Acc1 are epistatic, not additive, and work within the same pathway. If the mechanism through which *eaf7Δ* cells suppress GD-SGs was only through Acc1 (or downstream pathways) and increased acetyl-CoA levels, one would predict that doxycycline treated *tet0*_*7*_*-ACC1* cells should display similar reductions in GD-SGs as *eaf7*Δ cells (untreated *eaf7*Δ*tet0*_*7*_*-ACC1* cells), as both have similar levels of acetyl-CoA. Instead we found that GD-SGs in doxycycline treated *tet0*_*7*_*-ACC1* cells is significantly higher than *eaf7*Δ cells (untreated *eaf7*Δ *tet0*_*7*_*-ACC1* cells) (Fig 8F). Nor did we detect a significant change in the percentage of cells with GD-SGs between e*af7*Δ (untreated *eaf7*Δ*tet0*_*7*_*-ACC1* cells) and *eaf7Δacc1* cells (doxycycline treated *eaf7*Δ *tet0*_*7*_*-ACC1* cells). Taken together the non-additive impacts on acetyl-CoA levels suggest that Eaf7 and Acc1 are epistatic and that *eaf7Δ* mutants are suppressing GD-SGs through increased cellular acetyl-CoA levels and a yet to be described secondary pathway.

### Acc1 activity is reduced in *eaf1Δ* cells

**Fig 9:**
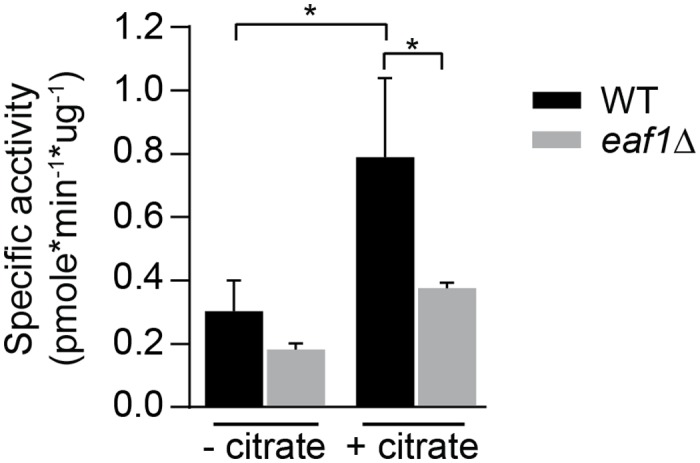
Acc1 activity is reduced in *eaf1Δ* cell extracts. WT (YKB33954) and *eaf1*Δ (YKB3929) cells grown at 30°C in YPD medium and cell lysates from exponential-phase cells were analyzed for acetyl-CoA carboxylase activity and specific activity was calculated as described in Materials and Methods. Three biological replicates and three technical replicates per biological replicate were performed. Error bars represent Mean ± SD and stars indicate statistical significance based on ANOVA and Turkey’s post hoc test (p value < 0.05).

To test the possibility that NuA4 modulates acetyl-CoA level through regulation of Acc1, we performed an Acc1 activity assay on whole cell extracts from wild type and the NuA4 deletion mutant *eaf1*Δ cells. As expected Acc1 activity detected in the whole cell extracts is increased upon the addition of citrate (Fig 9) [63, 64]. Though protein levels of Acc1 are not impacted by presence or absence of Eaf1 (S7 Fig), we found that Acc1 activity is significantly decreased in *eaf1Δ* cells (Fig 9). The reduction in Acc1 activity in *eaf1Δ* suggests that NuA4 is required for full Acc1 activity and provides insight into the mechanism by which NuA4 mutants display increased acetyl-CoA levels and suppression of GD-SG formation.

## Discussion

Here we demonstrate that NuA4 KAT function is required for SG formation upon glucose deprivation (Figs 1 and 2, S2 Fig and S2 Table), and this is partially mediated through the regulation of the metabolite acetyl-CoA (Fig 8). While it could be argued that the impacts of Eaf7/5/3 on GD-SGs could be through their non-NuA4 role as part of the TINTIN complex [reviewed in 65], this is unlikely as defects in GD-SG formation were also found in the *eaf1*Δ cells and temperature sensitive *esa1-ts* allele. Furthermore, as seen for many phenotypes associated with NuA4 mutants [45, 49, 66], GD-SG defects displayed by the scaffold mutant *eaf1*Δ are more pronounced than those of TINTIN mutants (Fig 2). We determined that NuA4 mutant *eaf7Δ* possess a delay in SG assembly rather than a complete inhibition of GD-SG formation (Fig 3). As *eaf7Δ* cells display minimal fitness defects, this result suggests that the delay in SG formation is not a reflection of sickness or cell cycle defects. Rather as *eaf7Δgcn5Δ* double mutant shows a significant reduction in GD-SGs throughout the 60 minute glucose deprivation time course (Fig 3B), it suggests that while NuA4 is the primary KAT regulating GD-SG formation that in its absence Gcn5 can partially compensate.

How is NuA4 regulating formation of SGs upon glucose deprivation? Given the role of NuA4 in histone acetylation and transcription, SG defects of NuA4 mutants could reflect defects in mRNA expression and in turn protein levels of structural SG proteins like Pab1 or Pub1. Despite the fact that deletion of *EAF1* reduces Pab1-GFP and Pub1-GFP protein levels while deletion of *EAF7* has no effect (S1 Fig), both *eaf1*Δ and *eaf7Δ* cells have significant reduction in GD-SG formation. This suggests that NuA4-dependent regulation of at least Pab1 or Pub1 protein levels have minor contribution to GD-SG dynamics. Indeed, most mutants identified in genome-wide screens with defects in SGs do not impact Pab1 protein levels, suggesting regulation of protein abundance is likely not a common mechanism for SG regulation [10, 67]. Further, we eliminate two other likely candidate mechanisms through which NuA4 could be regulating GD-SG, inhibition of translation and AMPK1/Snf1 (Fig 5). The later finding is particularly unexpected given that NuA4 inhibits Snf1 activity [41]. Neither a mutant that had elevated Snf1 activity, *hxk2*Δ [55], or deletion of *SNF1* itself, impacted GD-SG formation as assessed by the Pab1-GFP SG marker. Though it has been proposed, but not tested, that Snf1-dependent activation of PAS kinase Psk1 is required for the incorporation of Ppb1 into GD-SGs [23], our results suggest this might not be the case. One possibility is signaling pathways other than Snf1 are activating and regulating Psk1. Alternatively Snf1 and Psk1 may contribute to other aspects of SG dynamics at a later time point. Indeed, our study assessed SGs at 10 minutes glucose deprivation, while the Psk1 study assessed SGs after 60 minutes, a time point where we see resolution of SGs (Fig 3). Similarly, the role of AMPK in mammalian SG dynamics is also conflicted. While AMPK1 is required for cold shock induced SG formation in COS7 cells [68], in HeLa cells increased AMPK1 activity does not induce SGs, but pre-activation of AMPK1 prior to diethyl maleate treatment causes smaller and more SGs [69]. Clearly further studies will be needed to fully assess the role of Snf1/AMPK in SG dynamics.

Our work suggests that NuA4 is regulating GD-SG formation through both an acetyl-CoA dependent and acetyl-CoA independent pathways. Surprisingly we found that NuA4 and Gcn5 are not just mediating the downstream effects of acetyl-CoA oscillations, but rather that these KATs also influence acetyl-CoA levels. Genetic and chemical manipulations that increase or decrease acetyl-CoA levels led to the suppression or induction of GD-SGs (Figs 6–8). Similar to the ability of NuA4 mutants to only suppress GD-SG formation, acetyl-CoA suppression of SG formation is also likely stress specific as exogenous acetate treatment could not suppress heat-shock (Fig 6D) or ethanol (S2D Fig) induced SG formation. Furthermore our work suggests that acetyl-CoA is not the only driver of GD-SG formation. Reduction of acetyl-CoA levels in *acs1Δ* cells increase the percentage of cells with SGs upon glucose deprivation, but not under glucose replete conditions (Fig 8C). This suggests that decreases in acetyl-CoA alone is not enough to drive SG formation, but can escalate formation upon glucose deprivation. We found that while deletion of *ACS1* elevated the number of cells that formed SGs upon glucose deprivation (Fig 8C), deletion of *ACS2* had no influence on GD-SG formation (S6 Fig) which was surprising given that Acs2 is the nucleoctyosolic acetyl-CoA synthases essential for production of acetyl-coA on glucose [30]. In contrast, the cellular function of Acs1 is not as clear. While Acs1 has been suggested to be a mitochondrial acetyl-CoA synthase [70], Acs1 has been localized to the cytoplasm and nucleus [71, 72]. Further Acs1 is required for acetyl-CoA production on non-fermentable carbon sources, even in the absence of *ACS2* [62], indicating that Acs1 function is not limited to the mitochondria but also contributes to nucleocytosolic acetyl-CoA regulation. As *ACS1* is transcriptionally induced upon glucose depletion [60, 61], it is possible that Acs1-dependent generated acetyl-CoA upon glucose deprivation is required to attenuate or for the resolution of GD-SGs. While we cannot exclude the possibility that other metabolites derived from acetyl-CoA are responsible for suppression of GD-SGs, the genetics suggest that acetyl-CoA is a regulator of the GD-SG response.

How is acetyl-CoA supressing glucose-deprived SGs? A tempting scenario is that upon glucose deprivation nucleocytosolic acetyl-CoA depletion contributes to SG formation by a yet-to-be-characterized mechanism, and that mutations or treatments that increase nucleocytosolic acetyl-CoA, such as exogenous acetate treatment (Fig 6), reduction in Acc1 (Fig 7) or deletion of *EAF7* (Fig 8), mask this pathway. Though nucleocytosolic acetyl-CoA levels are a sensitive gauge for the metabolic state of the cell, high during glucose rich growth and low during fasted or carbon-poor states [reviewed in 34], the extent and timing of acetyl-CoA oscillations upon acute glucose deprivation has yet to be studied. Given that exogenous acetate can be converted to acetyl-CoA and then metabolised within minutes even in yeast cells in which the growth rate is significantly reduced [28], detailed flux analysis of nucleocytosolic acetyl-CoA upon stresses such as glucose deprivation remains a challenge to measure. However the idea that increased acetyl-CoA can suppress a stress response is not unique. Increased nucleocytosolic acetyl-CoA levels suppress autophagy through histone acetylation and repression of autophagy genes in both human and yeast cells [37, 38]. Could similar events be occurring to suppress GD-SG formation? While transcriptional events maybe contributing to SG dynamics, the rapid assembly of GD-SGs and the fact that SG assembly can occur in the absence of translation suggest that the acetylation state of non-histone proteins maybe a more likely mechanism regulating GD-SG assembly. Hence if the speculated model is correct and acetyl-CoA pools rapidly decrease upon glucose deprivation, one potential model is that under glucose replete “normal” acetyl-CoA levels KAT(s) are acetylating a target(s) that suppress SG formation. Indeed lysine acetylation has been detected on SG subunits Pbp1, Pub1 and Pab1 under glucose replete conditions [73–75], however it has yet to be determine if changes in glucose impact the acetylation status of these sites and if these lysine acetylations impact SG formation. Direct acetylation of SG proteins by NuA4 could also be the secondary molecular mechanism through which NuA4 is regulating GD-SGs distinct from the regulation of acetyl-CoA levels (Fig 8F). We also cannot exclude the model that acetylation of SG subunits are promoting GD-SG formation as seen for acetylation of TDP-43 that promotes its aggregation into stress granules [24]. Given the depth and dynamic nature of lysine acetylation that is occurring in the cell, a likely scenario is that lysine acetylation controls multiple aspects of SG dynamics.

If acetyl-CoA is suppressing GD-SG formation through hyper-activation of a KAT, our work suggests it is not through either NuA4 or Gcn5. Exogenous acetate does not require Eaf7 or Gcn5 to suppress GD-SG formation (Fig 6F) and elevating acetyl-CoA levels by decreasing Acc1 protein levels, still suppresses GD-SGs even in *eaf7*Δ cells (Fig 8F). Furthermore, if suppression was through a KAT, one would predict its deletion would result in increased GD-SG formation. However, our screen of known and putative non-essential KATs did not identify such a mutant (S2 Table), albeit the essential KAT *ECO1* was not screened and functional redundancies of the KATs may also be hindering identification. As proposed above, if KATs and KDACs regulate multiple aspects of SG dynamics, including promotion, maintenance and disassembly, our simple KAT/KDAC screen would not suffice and will require detailed kinetics or identification of specific acetylation sites. Alternatively, the impact of acetyl-CoA on GD-SGs may be occurring independent of KATs. Non-enzymatic acetylation of proteins occurs widely [75] and increased acetyl-CoA could led to acetylation of exposed lysine charge patches influencing aggregation of SG proteins directly or acetylation of yet-to-be-identified pathways contributing to GD-SG formation. Acetyl-CoA is also required for a wide variety of enzymes and pathways including the stimulation of KDAC activity [76]. As KDAC mutants display increased number of SGs even under non-stress conditions (S2 Table and [10]), acetyl-CoA activation of KDACs may contribute to the mechanism(s) by which acetyl-CoA delicately balances GD-SGs. Given the plethora of acetylation sites on SG proteins and the breath of enzymes/pathways regulated by acetyl-CoA, likely multiple mechanisms are mediating acetyl-CoA’s role in GD-SG dynamics.

Further this work challenges the paradigm that KATs are only mediating the effects of acetyl-CoA through acetylation of histones and other substrates. Rather our work implicates NuA4 as regulating acetyl-CoA level through Acc1 the rate-limiting enzyme for de novo fatty acid synthesis (Figs 8 and 9). The additive effects of *eaf7*Δ and *gcn5*Δ potentially suggest that each KAT is regulates acetyl-CoA levels through distinct mechanism or they have a shared substrate that regulates acetyl-CoA levels, and in the absence of one KAT, the other is able to partially compensate. The first scenario is the most likely as *ACC1* transcription is decreased in *gcn5* cells [77], but transcriptome studies have not detected a role NuA4 in regulation of the gene expression of *ACC1* [78, 79] nor do we detect an impact on Acc1 protein levels in *eaf1Δ* or *eaf7Δ* cells (S7 Fig). How is NuA4 regulating Acc1? Acetylome studies have determined that Acc1 is heavily acetylated in both human and yeast cells and that acetylation changes upon stress [73–75, 80, 81]. Nor is acetylation limited to Acc1, but sites were detected on all proteins of the Fatty Acid Synthases (FAS) pathway, suggesting that lipogenesis could be directly regulated by acetylation of enzymes. Future studies will need to be conducted to determine whether proteins of the FAS pathway, including Acc1, are regulated by NuA4-dependent acetylation.

In conclusion, our study not only identifies NuA4 as a regulator of GD-SG assembly in yeast, but identifies the central metabolite acetyl-CoA as a rheostat to fine-tune GD-SG dynamics. Though the exact mechanism by which NuA4 and acetyl-CoA regulate GD-SGs remains unclear, our work suggests that NuA4 is not acting solely downstream of acetyl-CoA, but contributes to regulating acetyl-CoA levels through Acc1. The complex cross-talk between NuA4 and acetyl-CoA maybe critical to link glucose availability to the cellular stress response.

## Materials and methods

### Yeast strains, plasmids and growth conditions

*S*. *cerevisiae* strains and plasmid used in this study are listed in S2 Table. Genetic deletion or epitope tag integrations were generated using standard PCR-mediated techniques as previously described [82], and validated by PCR with sequence-specific primers and/or Western Blot. Yeast cultures were grown at 30°C unless otherwise indicated with constant shaking at 200 rpm in standard YPD medium (1% yeast extract, 2% peptone, 2% dextrose) or synthetic complete SCD medium (0.67% yeast nitrogen base without amino acids, 0.2% amino acid drop out mix, 2% dextrose).

### Yeast microscopy and image quantification

Glucose deprivation, acetate and heat-shock stresses were conducted as described previously [13, 28, 83]. Briefly, yeast strains were grown in the appropriate media overnight before dilution to an OD_600_ of 0.1 and grown to mid-logarithmic growth (OD_600_ of 0.5–0.8) prior to treatment. For glucose depletion and control experiments, cells were collected by centrifugation (3 min at 3,000 rpm at 30°C), washed in 30°C pre-warmed medium with or without glucose, re-suspended in either glucose or glucose-deprived medium, and then incubated with constant shaking for 10 mins unless otherwise indicated. For acetate treatment, acetate (2.5M Acetate Solution, Sigma-Aldrich; cat. # 3863) was added to re-suspension media to a final concentration of 100 mM for 10 min. For heat-shock stress, yeast cultures were collected by centrifugation (3 min at 3,000 rpm at 30°C), re-suspended in pre-warmed medium at 46°C, and then incubated at 46°C for 10 min. For ethanol stress, ethanol was added to exponentially growing cells to a final concentration of 15% for 10 min. Similar to acetate suppression of glucose deprivation, 2% ethanol, 2% citrate (sodium citrate dihydrate: Fisher Scientific, S279-3) or 2% pyruvate (sodium pyruvate, Sigma-Aldrich, cat# P2256) was added to glucose-deprived medium for 10 min. For the yeast strains carrying the tetracycline-repressible *tet07* promoter, doxycycline (Sigma-Aldrich; cat. # D3447) was added to exponentially growing cells to a final concentration of 10 uM for 2.5 h [59] prior to stress treatment and doxycycline exposure was maintained throughout the experiment. After stress treatment, 1 ml of yeast cells was collected by centrifugation (3 min at 3,000 rpm at room temperature), re-suspended in 20 μl of pre-warmed SC medium to maintain stress (example SC +/- glucose), prior to immediate microscopic examination in absence of fixative agents.

A Leica fluorescence microscope (DMI6000; Leica Microsystems) equipped with a high-performance camera (Hamamatsu), DG4 light source (Sutter Instruments) and Volocity 4.3.2 software (PerkinElmer) was used for all of the imaging acquisition, processing, including blind quantification scoring of the SGs [84]. All experimental images were captured as Z-stacks (0.2 μm steps across 6 μm), which were collapsed and used for manual image quantification in a blind manner. A least three independent biological replicates for each yeast strain and condition were performed with 100 cells/replicate scored for SGs (% of cells with SGs) and 50 cells/replicate scored for the number of SGs/cell. From these values, the percentage of cells that form SGs was determined, and statistical significance below the p-value of 0.05 was measured using two-way ANOVA or unpaired t-tests.

### Quantitative western blot analysis

As indicated in text, whole cell extracts (WCE) were prepared using either the modified immunoprecipitation (mChIP) [85] or Trichloroacetic acid (TCA) [86] protocols. Protein concentration of the mChIP WCE was measured by using Bradford reagent (Bio-Rad; cat. # 500–0006) according to the manufacturer’s instructions. Equal numbers of cells were used for TCA WCE and equal volumes of WCE were utilized for Western Blot analysis. Proteins were separated by SDS-PAGE gels and electrophoretically transferred to nitrocellulose membranes (PALL; cat. # 66485). Membranes were incubated for 60 min at room temperature (RT) in blocking buffer (PBST-5% milk), prior to incubation overnight at 4°C with the primary antibody diluted in blocking buffer (PBST-5% milk). The immunoblots were washed three times in PBST and incubated with the secondary antibody for 2 h at RT in blocking buffer (PBST-5% milk). The immunoblots were washed again three times in PBST, and the proteins were visualized by chemiluminescence (Clarity ECL substrate, Bio-Rad). The following dilutions of primary antibodies were used: 1:5,000 mouse monoclonal anti-GFP (Sigma-Aldrich; cat. # 11814460001); 1:1,000 rabbit polyclonal anti-hyperacetylated histone H4 (EMD Millipore; cat. # 06–946); 1:1,000 rabbit monoclonal anti-histone H3 (EMD Millipore; cat. # 05–928); 1:1,000 rabbit polyclonal anti-Acc1 (Cell Signaling; cat. # 3662); 1:10,000 rabbit polyclonal anti-G6PDH (Sigma-Aldrich; cat. # A9521). The following dilutions of secondary antibodies were used: 1:5,000 goat-anti-mouse horseradish peroxidase (HRP)-conjugated (BioRad; cat. # 170–6516) and 1:5,000 goat-anti-rabbit HRP-conjugated (Chemicon; cat. # AP307P).

As indicated, protein level quantification was performed using either standard densitometry with an internal protein control or the entire lane signal using the TGX Stain-Free FastCast Acrylamide Kit (Bio-Rad; cat. # 161–0181) as previously described [87, 88]. Statistical significance below the p-value of 0.05 was measured using ANOVA or unpaired t-tests.

### Analysis of ribosomal distribution on sucrose density gradients

Briefly, 50 ml of yeast cells cultured in glucose or subjected to glucose deprivation stress for 10 min were harvested by centrifugation (3 min at 3,000 rpm at 4°C) in the presence of cycloheximide (Sigma-Aldrich; cat. # C4859) at the final concentration of 100 μg/ml. The cells were washed once in cold distilled water containing 100 μg/ml cycloheximide, and cell pellets were stored at -80°C. Ribosomal distribution was then performed as previously described [89]. 10 A_260_ units diluted in 400 μl of lysis buffer were loaded onto 15–45% linear sucrose gradients. The gradients were centrifuged (90 min at 39,000 rpm) in a SW41 rotor (Beckman Instruments), after which the gradients were collected from the top with a gradient fractionation system (Brandel). The A_254_ was measured continuously to generate traces.

### HeLa and MCF7 cell culture conditions and immunofluorescence

HeLa and MCF7 cell lines were purchased from the American Type Cell Collection (ATCC). HeLa cells were grown and maintained in Dulbecco’s modified Eagle’s Medium (DMEM) supplemented with 2 mM L-glutamine and 10% fetal bovine serum. MCF7 cells were grown in DMEM medium, but supplemented with 2.75 μg/ml insulin. HeLa and MCF7 cells were treated with the following drugs: NU9056 (Tocris; cat. # 4903) was diluted in DMSO and used at a final concentration of 20 μM for 24 h; Bortezomib (LC Laboratories; cat. # B-1408) was diluted in DMSO and used at a concentration of 10 μM for 4 h; Sodium arsenite (Sigma-Aldrich; cat. # S7400) was diluted in Phosphate Buffer Saline (PBS) and used at a final concentration of 100 μM for 60 min. Immunofluorescence was then performed on the cells at the end of the specific drug treatment. Cells were washed three times with 1X PBS, fixed in 4% paraformaldehyde, washed again three times with 1X PBS, and permeabilized with 0.5% Triton X-100 in 1X PBS. Cells were washed three times, incubated with the primary antibody for 60 min, then washed once with 0.1% Triton X-100 in 1X PBS, and washed twice with 1X PBS. Cells were incubated with Alexa Fluor secondary antibody for 60 min, and washes were done similarly as after the incubation with the primary antibody. Cells were mounted on slides using Vectashield Hard Set Mounting Media (Vector Labs). Fluorescence microscopy was performed using the Zeiss Axio Imager Z1 microscope, and images were acquired with an AxioCam HR camera utilizing Zeiss Axiovision 4.5 software. The following primary antibodies were used: anti-FMRP (1:100; EMD Millipore; cat. # MAB2160); anti-DDX3 (1:100; Bethyl Laboratories; cat. # A300-474A); anti-TIA-1 (1:50; SantaCruz Biotechnology; cat. # SC-1751). The following secondary antibodies (Life Technologies) were used: rabbit (1:200; cat. # A110012), mouse (1:200; cat. # A11005), and goat (1:100; cat. # A11058) Alexa Fluor 594 nm antibodies.

### Histone preparation of HeLa and MCF7 cells and analysis

Cells were washed twice with cold 1X PBS and lysed for 10 min in TEB buffer (0.5% Triton X-100; 2 mM phenylmethylsulfonyl fluoride, 0.02% sodium azide in 1X PBS). Nuclei were pelleted by centrifugation (10 min at 6,500 g at 4°C), washed once in TEB buffer, and centrifuged again. The pellet was resuspended in 0.2 N hydrochloric acid in 1X PBS, and histones were extracted overnight at 4°C, then centrifuged (10 min at 6,500 g at 4°C). Protein concentration was measured by Bradford reagent, and histones were resolved by SDS-PAGE. Proteins were transferred to Immunobilon-P polyvinylidene difluoride membranes (PVDF; EMD Millipore). Membranes were incubated for 60 min at RT in blocking buffer (PBST-5% milk). Membranes were then incubated overnight at 4°C with the primary antibody diluted in blocking buffer (PBST-2% milk). The immunoblots were washed three times in PBST and incubated with the secondary antibody for 60 min at RT in blocking buffer (PBST-2% milk). The immunoblots were washed again three times in PBST, and the proteins were visualized by using the chemiluminescence detection kit (Luminata ECL substrate, EMD Millipore). The following primary antibodies and dilutions were used: 1:1000 rabbit polyclonal anti-hyperacetylated histone H4 (EMD Millipore; cat. # 06–946); 1:10,000 mouse monoclonal anti-Tubulin (Sigma-Aldrich; cat. # T6199). The following secondary antibodies were used: 1:20,000 goat anti-mouse HRP-conjugated (Cappel; cat. # 55479); 1:20,000 goat anti-rabbit HRP-conjugated (Cappel; cat. # 55690).

### Acetyl-CoA extraction and measurements

50 ml of yeast cell culture were harvested and immediately processed for acetyl-CoA extraction as previously described [90]. Acetyl-CoA level was measured using an Acetyl-Coenzyme A assay kit (Sigma-Aldrich; cat. # MAK039) according to the manufacturer’s instructions. A SynergyH1 Multi-Mode Plate Reader (BioTek) was used to measure the fluorometric product (λ_ex5_ = 535/λ_em_ = 587) of each assay reaction.

### Acetyl-CoA carboxylase assay

Acetyl-CoA carboxylase assay was performed similar to previously described [64] with adapted for yeast extraction. Exponentially growing cells were lysed in lysis buffer (100 mM Hepes pH 8.0, 20 mM MgOAc, 300 mM NaOAc, 10% Glycerol, 10 mM EGTA, 0.1mM EDTA and 1% NP-40) supplemented with mini protease inhibitor cocktail tablet (Roche; cat. # 4693159001), KDAC inhibitors (50 mM nicotinamide, 50mM sodium butyrate, 0.4μM apicidin (Sigma; cat. # EPI008-1KT), 2μM M344 (Sigma; cat. # EPI008-1KT), 250μM splitomycin (Sigma; cat. # EPI008-1KT), 5μM TSA (Sigma; cat. # EPI008-1KT), 10mM Sodium 4-phenylbutyrate (Sigma; cat. # EPI008-1KT) and 1mM valproic sodium salt (Sigma; cat. # EPI008-1KT)) and phosphatase inhibitors (5mM NaF, 1mM Na_3_VO_4_, phosphatase inhibitor cocktail 2 and 3 (Sigma; cat. # P5726 and P0044, respectively)) by bead beating. Cell lysates were obtained by centrifugation at 16,000 x g for 15 min at 4°C. Acc1 assay was performed by incubating equal amounts of cell lysates with reaction buffer (50 mM Hepes pH 7.4, 10mM MgCl_2_, 1 mM MnCl_2_, 2 mM DTT, 0.4 mM ATP, 0.075% fatty acid free BSA (Sigma; cat. # A7030), 12.5 mM NaHCO_3_ and 1.5 μCi ^14^C-NaHCO_3_ (Perkin Elmer; cat. # NEC086H005MC) with or without 10 mM citrate. The reactions were incubated at room temperature for 90 min, stopped by the addition of 0.6 N HCl and air-dried overnight at 37°C. The radioactivity of the products was determined by scintillation counting of the dried materials. Specific activity was calculated as the amount of substrate conversion divided by 90 min and the amount of total protein in the lysate as determined by microBCA kit (Thermo Scientific; cat. # 23231). ANOVA and Turkey’s post hoc test were performed with data from 3 biological replicates, each of which has 3 technical replicates.

## Supporting information

S1 Fig NuA4 regulates glucose-deprived stress granule assembly independently of Pab1 protein level. **A**) Pab1-GFP protein levels are decreased in *eaf1*Δ cells, but not in *eaf7Δ* cells. WT (YKB3114), *eaf1*Δ (YKB3382) and *eaf7*Δ (YKB3336) cells expressing endogenously tagged Pab1-GFP were grown to mid-log phase at 30°C in YPD medium (+glucose), subjected to glucose deprivation (-glucose) for 10 minutes. mChIP protein extraction was performed and 30 μg of whole cell extract was resolved by SDS-PAGE prior to Western Blot analysis using an antibody against GFP. Graph displays the average of Pab1-GFP protein level to WT glucose conditions of three independent experiments +/- the SEM. **B**) NuA4 does not significantly change Pub1-GFP protein level. WT (YKB3115), *eaf1*Δ (YKB3339) and *eaf7*Δ (YKB3337) cells expressing endogenously tagged Pub1-GFP were grown to mid-log phase at 30°C in YPD medium, subjected to glucose deprivation for 10 minutes. mChIP protein extraction was performed and 40 μg of the protein extract was resolved by SDS-PAGE prior to Western Blot analysis using an antibody against GFP. Graph displays the average of Pub1-GFP protein level to WT glucose conditions of three independent experiments +/- the SEM. * denotes statistical significance at a p-Value < 0.05 determined using a two-way ANOVA test. Error bars indicate SEM.
(TIF)

S2 Fig Eaf7 is not required for heat-shock or ethanol induced stress granules. **A-C**) WT (YKB3114), *eaf7*Δ (YKB3336), *eaf1*Δ (YKB3382), *gcn5*Δ (YKB4116), and *eaf7*Δ*gcn5*Δ (YKB4118) cells expressing endogenously tagged Pab1-GFP were grown to mid-log phase at 30°C in YPD medium (no heat-shock) and then subjected to heat-shock at 46°C for 10 minutes. **A**) Representative florescent images. Red scale bar: 5 μm. **B**) Quantification of percentage of cells with Pab1-GFP foci. Results are the average of three biological replicates, a minimum of 100 cells per replicate were scored, error bars indicate the standard error of the mean (SEM). * denotes statistical significance at a p-Value < 0.05 determined using two-way ANOVA. **C**) *eaf7*Δ*gcn5*Δ cells display minimal growth defects. WT (YKB3114), *eaf7*Δ (YKB3336), *gcn5*Δ (YKB4116), and *eaf7*Δ*gcn5*Δ (YKB4118) cells expressing endogenously tagged Pab1-GFP were plated in 10-fold serial dilutions (A_600_ = 0.1, 0.01, 0.001, 0.0001) onto YPD plates and incubated at 30°C for 3 days. Dot assay shown are from the same plate; white line indicates strains that were removed from image. **D-E)** WT (YKB3263) and *eaf7*Δ (YKB3729) cells transformed with pPAB1-GFP were grown to mid-log phase at 30°C in SC-URA and then ethanol was added to a final concentration of 15% for 10 minutes. **D)** Quantification of percentage of cells with Pab1-GFP foci. Results are the average of three biological replicates, a minimum of 100 cells per replicate were scored, error bars indicate the standard error of the mean (SEM). **E**) Representative florescent images. Red scale bar: 5 μm.
(TIF)

S3 Fig Representative florescent images for Fig 6. **A**) Images correlate to quantitation in Fig 6C. **B**) Images correlate to quantitation in Fig 6D. **C**) Images correlate to quantitation in Fig 6E. **D**) Images correlate to quantitation in Fig 6F. Red scale bar: 5 μm.
(TIF)

S4 Fig Ethanol treatment can partially suppress GD-SG formation. WT (YKB3114) cells expressing endogenously tagged Pab1-GFP were grown to mid-log phase at 30°C in YPD medium, and subjected to either glucose deprivation for 10 minutes or glucose deprivation plus either 100mM acetate, 2% galactose, 2% ethanol, 50mM citrate or 2% pyruvate for 10 minutes. **A**) Quantification of percentage of cells with Pab1-GFP foci. Results are the average of three biological replicates, a minimum of 100 cells per replicate were scored, error bars indicate the standard error of the mean (SEM). * denotes statistical significance at a p-Value < 0.05 determined using two-way ANOVA test. **B**) Representative florescent images. Red scale bar: 5 μm.
(TIF)

S5 Fig Representative florescent images for Figs 7 and 8. **A**) Images correlate to quantitation in Fig 7D. **B**) Images correlate to quantitation in Fig 8C. Red scale bar: 5 μm.
(TIF)

S6 Fig Acs2 does not impact glucose-deprived stress granule formation. **A-B**) Acs1-ts at semi-permissive temperature does not impact GD-SG formation, in contrast 1 hour incubation at 37°C inhibits GD-SG formation. Exponential-phase wild type (WT, YKB3114), *acs1Δ* (YKB4132), *acs2-ts* (YKB4022, [30]), and *acs2-ts acs1Δ* (YKB4121), cells expressing endogenously tagged *PAB1-GFP* grown in YPD (+glucose) at 25°C were either kept at 25°C or temperature shifted to 33°C or 37°C for 1 hour prior to 10 minutes of glucose deprivation. Cells were scored for SGs (Pab1-GFP foci). **A**) Quantitation of the percentage of cells with SGs. **B**) Representative florescent images. Red scale bar: 5 μm. **C-E**) Reduction of Acs2 protein level prior to glucose deprivation does not impact GD-SG formation. tet07-*ACS2* expressing endogenously tagged Pab1-GFP (YKB4246) were grown to mid-log phase at 30°C in YPD medium. Half of the culture was treated with 10 μM doxycycline for 2.5 hours prior to glucose deprivation for 10 minutes. **C**) Cells were scored for SGs. Quantification of the percentage of cells with SGs. **D**) TCA protein extraction was performed and whole cell extract (WCE) was resolved by SDS-PAGE prior to Western Blot analysis using the indicated antibodies. The image is representative of three replicates. **E**) Representative florescent images. Red scale bar: 5 μm. Results are the average of three biological replicates, a minimum of 100 cells per replicate were scored, error bars indicate the SEM. * denotes statistical significance at a p-Value < 0.05 determined using a two-way ANOVA test.
(TIF)

S7 Fig Acc1-GFP protein levels are not impacted by Eaf1 or Eaf7. Exponential-phase (+ glucose) wild type (WT, BY4741) or cells expressing endogenously tagged Acc1-GFP in WT, (YKB3954), *eaf7Δ* (YKB3930), and *eaf1Δ* (YKB3929) backgrounds were harvested both before and after 10 minutes of glucose deprivation (- glucose). TCA protein extraction was performed and whole cell extract (WCE) was resolved by SDS-PAGE prior to Western Blot analysis using the indicated antibodies. The image is representative of three experiments.
(TIF)

S1 Table Quantification of average foci per cells for glucose deprivation induced stress granules and P-bodies. A least three independent biological replicates for each yeast strain and condition were performed with 50 cells/replicate scored for the number of SGs/cell. From these values, the average foci per cell was determined; +/- standard error of the mean.
(DOCX)

S2 Table Systematic assessment of KAT and KDAC mutants for a role in glucose-deprived stress granule formation. Each mutant listed was transformed with *PAB1-GFP*::*URA*::*CEN* plasmid (pBK 192), cultured in SCD-URA medium (+glucose) at 30°C and exponential-phase cells were subjected to 10 minutes of glucose deprivation (-glucose) and immediately accessed for Pab1-GFP foci (SGs). Results are the average of the three biological replicates, a minimum of 100 cells per replicate were scored. Statistical significance at a p-Value < 0.05 was determined using an unpaired t-test.
(DOCX)

S3 Table Strains and plasmid used in this study. (DOCX)
