# Supplementary figures and images for: Lysine acetyltransferase NuA4 and acetyl-CoA regulate glucose-deprived stress granule formation in Saccharomyces cerevisiae
Source: PLoS Genet. 2017 Feb 23;13(2):e1006626. doi: 10.1371/journal.pgen.1006626 (PMC5344529; doi:10.1371/journal.pgen.1006626)

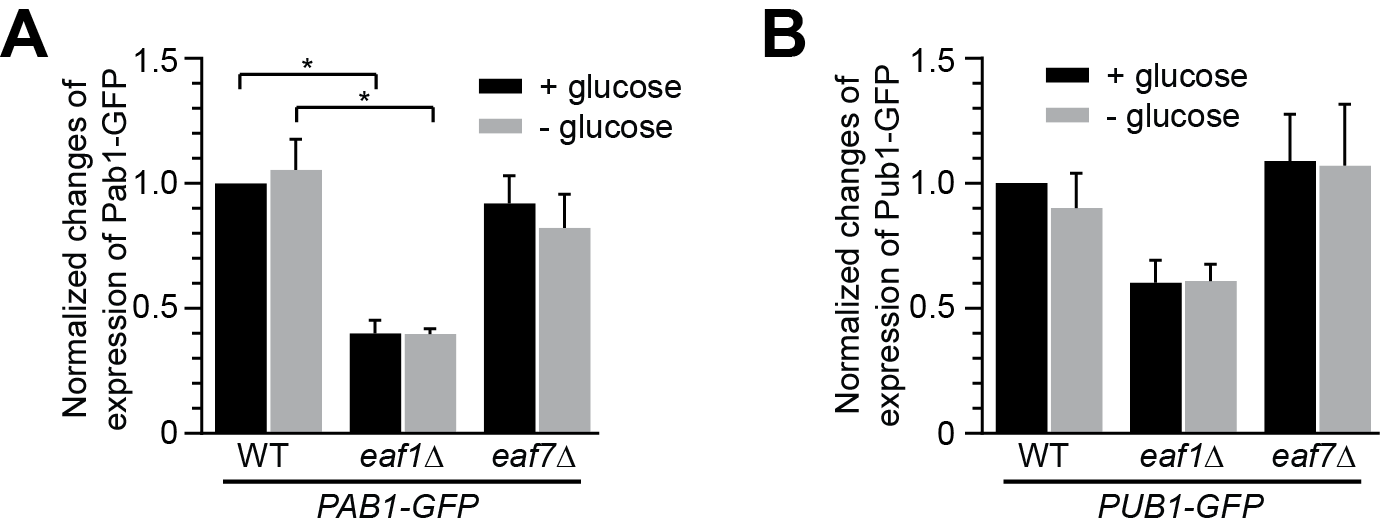

Supplement: S1 Fig — A) Pab1-GFP protein levels are decreased in eaf1Δ cells, but not in eaf7Δ cells. WT (YKB3114), eaf1Δ (YKB3382) and eaf7Δ (YKB3336) cells expressing endogenously tagged Pab1-GFP were grown to mid-log phase at 30°C in YPD medium (+glucose), subjected to glucose deprivation (-glucose) for 10 minutes. mChIP protein extraction was performed and 30 μg of whole cell extract was resolved by SDS-PAGE prior to Western Blot analysis using an antibody against GFP. Graph displays the average of Pab1-GFP protein level to WT glucose conditions of three independent experiments +/- the SEM. B) NuA4 does not significantly change Pub1-GFP protein level. WT (YKB3115), eaf1Δ (YKB3339) and eaf7Δ (YKB3337) cells expressing endogenously tagged Pub1-GFP were grown to mid-log phase at 30°C in YPD medium, subjected to glucose deprivation for 10 minutes. mChIP protein extraction was performed and 40 μg of the protein extract was resolved by SDS-PAGE prior to Western Blot analysis using an antibody against GFP. Graph displays the average of Pub1-GFP protein level to WT glucose conditions of three independent experiments +/- the SEM. * denotes statistical significance at a p-Value < 0.05 determined using a two-way ANOVA test. Error bars indicate SEM. (TIF) [file pgen.1006626.s001.tif]

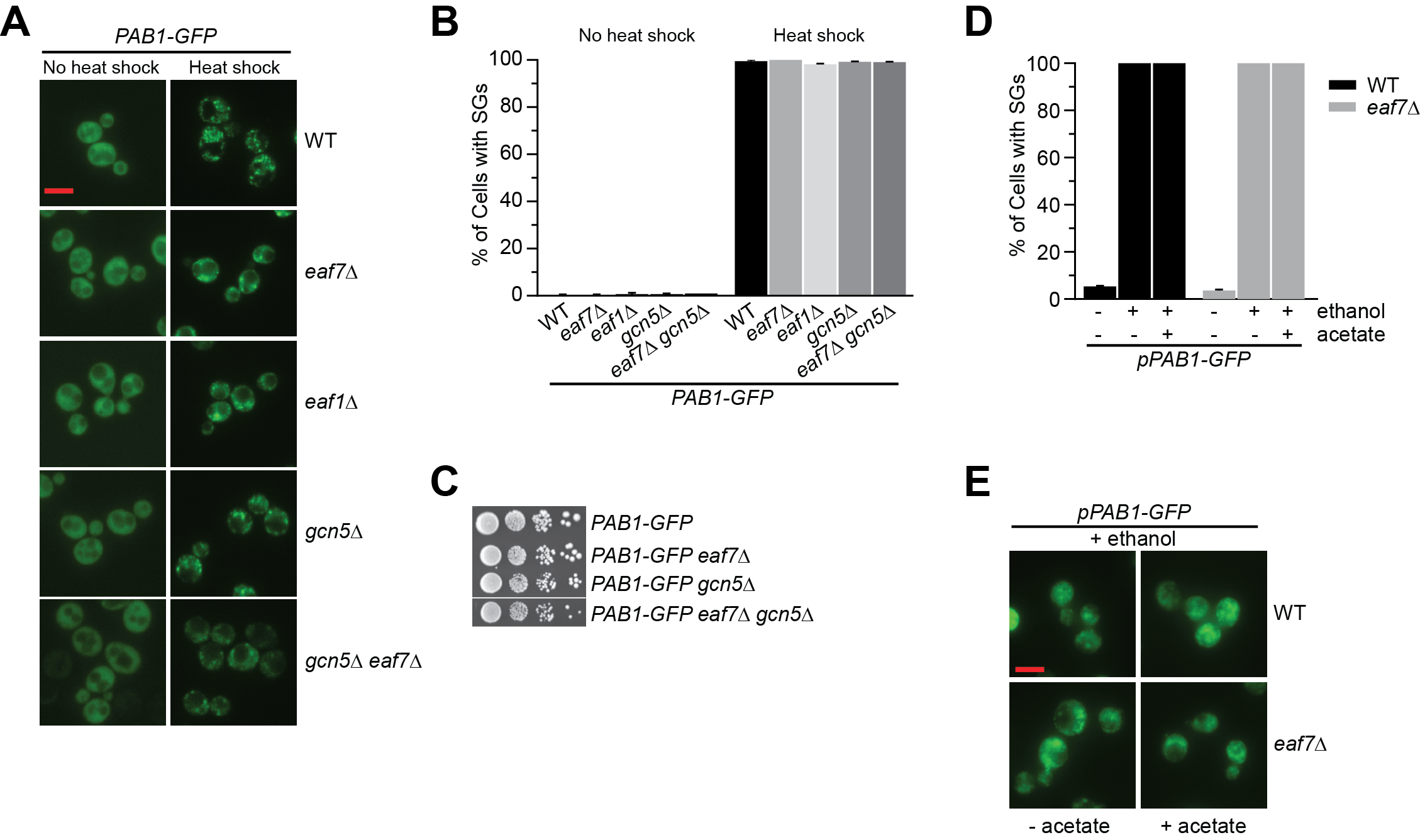

Supplement: S2 Fig — A-C) WT (YKB3114), eaf7Δ (YKB3336), eaf1Δ (YKB3382), gcn5Δ (YKB4116), and eaf7Δgcn5Δ (YKB4118) cells expressing endogenously tagged Pab1-GFP were grown to mid-log phase at 30°C in YPD medium (no heat-shock) and then subjected to heat-shock at 46°C for 10 minutes. A) Representative florescent images. Red scale bar: 5 μm. B) Quantification of percentage of cells with Pab1-GFP foci. Results are the average of three biological replicates, a minimum of 100 cells per replicate were scored, error bars indicate the standard error of the mean (SEM). * denotes statistical significance at a p-Value < 0.05 determined using two-way ANOVA. C) eaf7Δgcn5Δ cells display minimal growth defects. WT (YKB3114), eaf7Δ (YKB3336), gcn5Δ (YKB4116), and eaf7Δgcn5Δ (YKB4118) cells expressing endogenously tagged Pab1-GFP were plated in 10-fold serial dilutions (A600 = 0.1, 0.01, 0.001, 0.0001) onto YPD plates and incubated at 30°C for 3 days. Dot assay shown are from the same plate; white line indicates strains that were removed from image. D-E) WT (YKB3263) and eaf7Δ (YKB3729) cells transformed with pPAB1-GFP were grown to mid-log phase at 30°C in SC-URA and then ethanol was added to a final concentration of 15% for 10 minutes. D) Quantification of percentage of cells with Pab1-GFP foci. Results are the average of three biological replicates, a minimum of 100 cells per replicate were scored, error bars indicate the standard error of the mean (SEM). E) Representative florescent images. Red scale bar: 5 μm. (TIF) [file pgen.1006626.s002.tif]

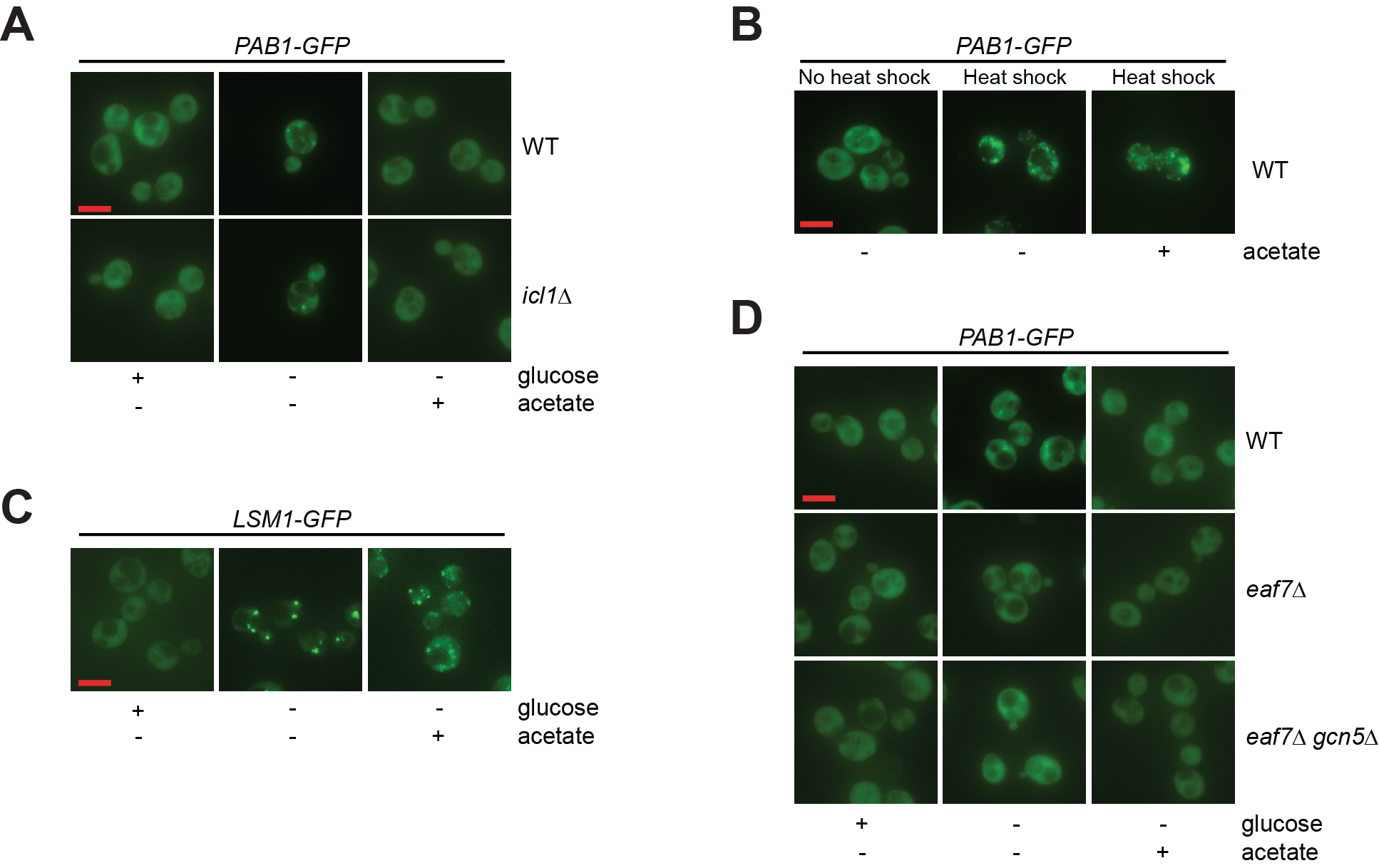

Supplement: S3 Fig — A) Images correlate to quantitation in Fig 6C. B) Images correlate to quantitation in Fig 6D. C) Images correlate to quantitation in Fig 6E. D) Images correlate to quantitation in Fig 6F. Red scale bar: 5 μm. (TIF) [file pgen.1006626.s003.tif]

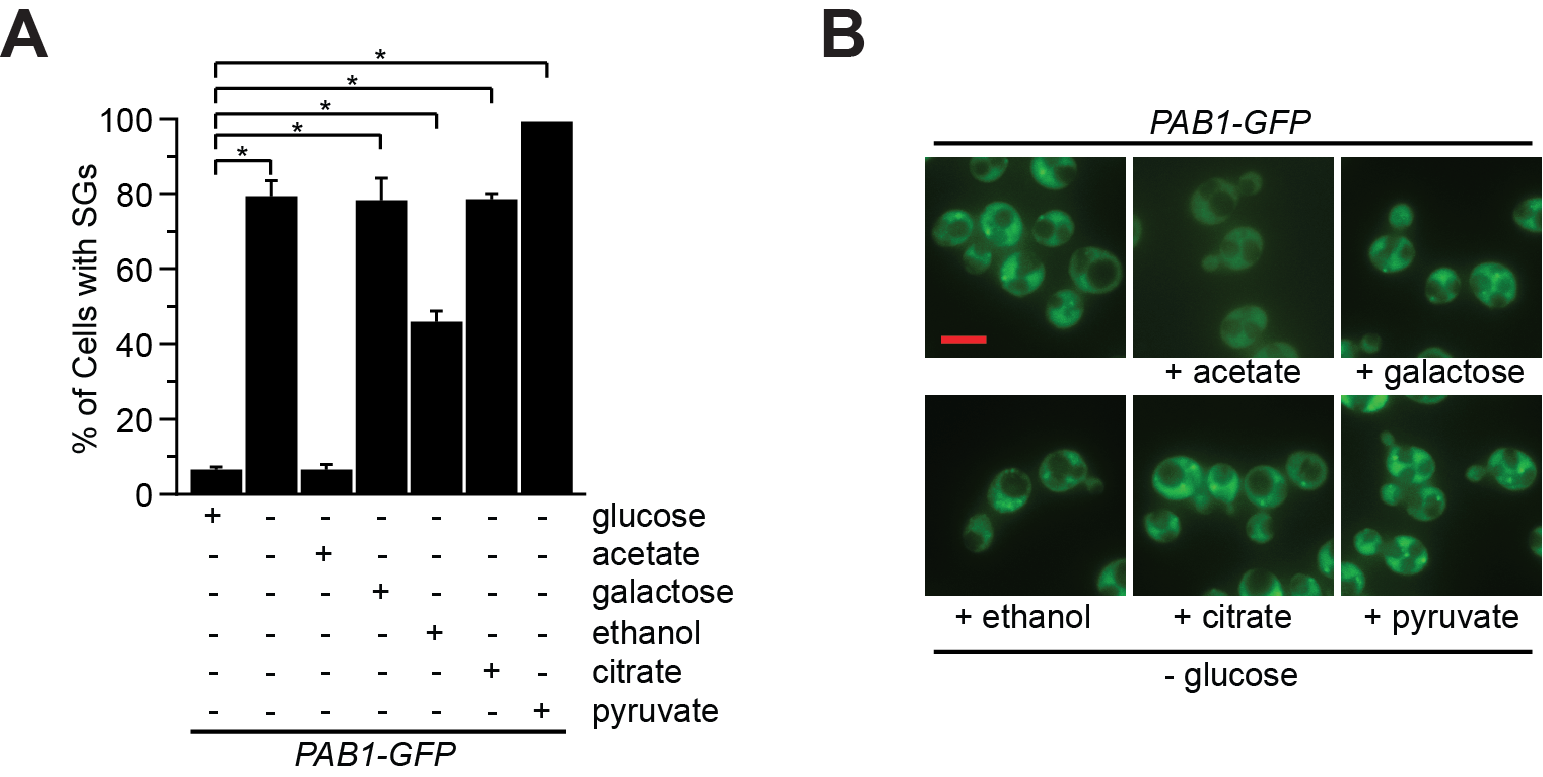

Supplement: S4 Fig — WT (YKB3114) cells expressing endogenously tagged Pab1-GFP were grown to mid-log phase at 30°C in YPD medium, and subjected to either glucose deprivation for 10 minutes or glucose deprivation plus either 100mM acetate, 2% galactose, 2% ethanol, 50mM citrate or 2% pyruvate for 10 minutes. A) Quantification of percentage of cells with Pab1-GFP foci. Results are the average of three biological replicates, a minimum of 100 cells per replicate were scored, error bars indicate the standard error of the mean (SEM). * denotes statistical significance at a p-Value < 0.05 determined using two-way ANOVA test. B) Representative florescent images. Red scale bar: 5 μm. (TIF) [file pgen.1006626.s004.tif]

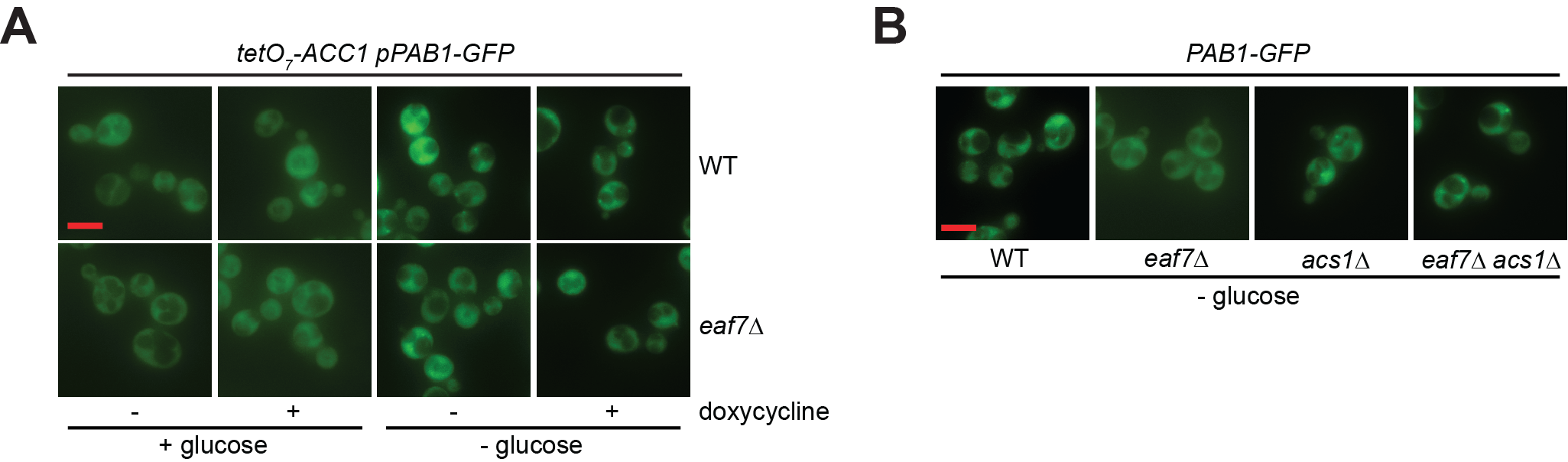

Supplement: S5 Fig — A) Images correlate to quantitation in Fig 7D. B) Images correlate to quantitation in Fig 8C. Red scale bar: 5 μm. (TIF) [file pgen.1006626.s005.tif]

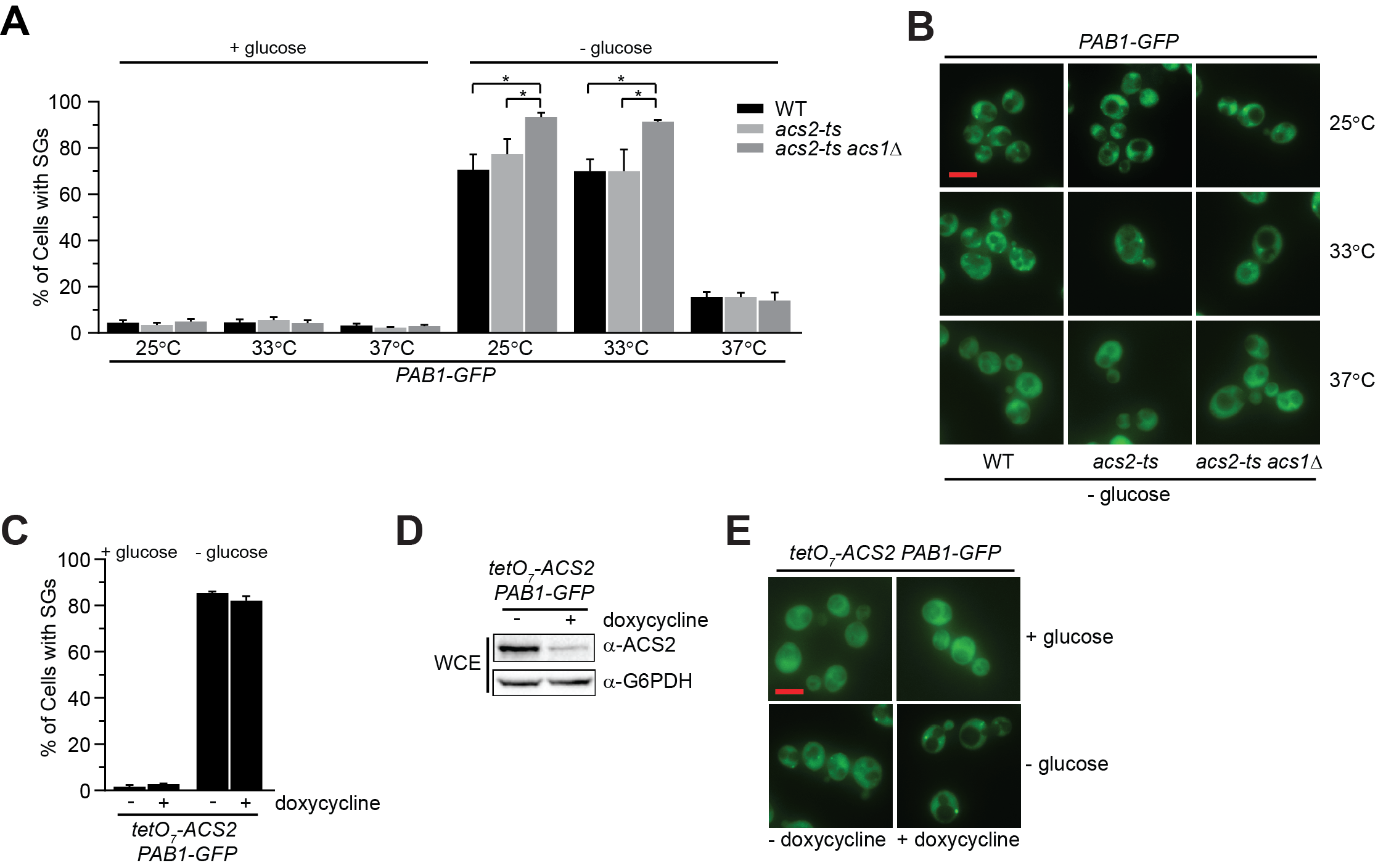

Supplement: S6 Fig — A-B) Acs1-ts at semi-permissive temperature does not impact GD-SG formation, in contrast 1 hour incubation at 37°C inhibits GD-SG formation. Exponential-phase wild type (WT, YKB3114), acs1Δ (YKB4132), acs2-ts (YKB4022, [30]), and acs2-ts acs1Δ (YKB4121), cells expressing endogenously tagged PAB1-GFP grown in YPD (+glucose) at 25°C were either kept at 25°C or temperature shifted to 33°C or 37°C for 1 hour prior to 10 minutes of glucose deprivation. Cells were scored for SGs (Pab1-GFP foci). A) Quantitation of the percentage of cells with SGs. B) Representative florescent images. Red scale bar: 5 μm. C-E) Reduction of Acs2 protein level prior to glucose deprivation does not impact GD-SG formation. tet07-ACS2 expressing endogenously tagged Pab1-GFP (YKB4246) were grown to mid-log phase at 30°C in YPD medium. Half of the culture was treated with 10 μM doxycycline for 2.5 hours prior to glucose deprivation for 10 minutes. C) Cells were scored for SGs. Quantification of the percentage of cells with SGs. D) TCA protein extraction was performed and whole cell extract (WCE) was resolved by SDS-PAGE prior to Western Blot analysis using the indicated antibodies. The image is representative of three replicates. E) Representative florescent images. Red scale bar: 5 μm. Results are the average of three biological replicates, a minimum of 100 cells per replicate were scored, error bars indicate the SEM. * denotes statistical significance at a p-Value < 0.05 determined using a two-way ANOVA test. (TIF) [file pgen.1006626.s006.tif]

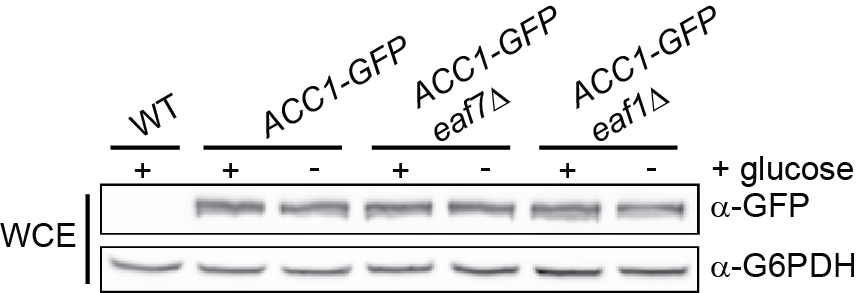

Supplement: S7 Fig — Exponential-phase (+ glucose) wild type (WT, BY4741) or cells expressing endogenously tagged Acc1-GFP in WT, (YKB3954), eaf7Δ (YKB3930), and eaf1Δ (YKB3929) backgrounds were harvested both before and after 10 minutes of glucose deprivation (- glucose). TCA protein extraction was performed and whole cell extract (WCE) was resolved by SDS-PAGE prior to Western Blot analysis using the indicated antibodies. The image is representative of three experiments. (TIF) [file pgen.1006626.s007.tif]
