# Supplementary material for: Lysine acetyltransferase NuA4 and acetyl-CoA regulate glucose-deprived stress granule formation in Saccharomyces cerevisiae
Source: PLoS Genet. 2017 Feb 23;13(2):e1006626. doi: 10.1371/journal.pgen.1006626 (PMC5344529; doi:10.1371/journal.pgen.1006626)
Supplement: S1 Table — A least three independent biological replicates for each yeast strain and condition were performed with 50 cells/replicate scored for the number of SGs/cell. From these values, the average foci per cell was determined; +/- standard error of the mean. (DOCX) [file pgen.1006626.s008.docx]

**S1 Table: Quantification of average foci per cells for glucose deprivation induced stress granules and P-bodies**

| Strain and condition | Average Foci/Cell | Strain and condition | Average Foci/Cell |
| --- | --- | --- | --- |
| + glucose FOCI (Stress granules) | | - glucose FOCI (Stress granules) | |
| BY4741 (WT) [empty vector] | 0 ± 0 | BY4741 (WT) [empty vector] | 0 ± 0 |
| BY4741 (WT) *[pPAB1-GFP]* | 0.05 ± 0.01 | BY4741 (WT) *[pPAB1-GFP]* | 1.97 ± 0.57 |
| *pbp1Δ* *[pPAB1-GFP]* | 0.03 ± 0.02 | *pbp1Δ* *[pPAB1-GFP]* | 1.15 ± 0.31 |
| *pub1Δ* *[pPAB1-GFP]* | 0.03 ± 0.02 | *pub1Δ* *[pPAB1-GFP]* | 2.69 ± 0.80 |
| *eaf1Δ* *[pPAB1-GFP]* | 0.03 ± 0.03 | *eaf1Δ* *[pPAB1-GFP]* | 0.43 ± 0.15 |
| *eaf7Δ* *[pPAB1-GFP]* | 0.06 ± 0.01 | *eaf7Δ* *[pPAB1-GFP]* | 0.75 ± 0.26 |
| *PAB1-GFP*  25°C | 0.05 ± 0.02 | *PAB1-GFP*  25°C | 0.69 ± 0.08 |
| *PAB1-GFP*  37°C | 0.03 ± 0.05 | *PAB1-GFP*  37°C | 0.48 ± 0.04 |
| *PAB1-GFP esa1-ts*  25°C | 0.01 ± 0.02 | *PAB1-GFP esa1-ts*  25°C | 0.76 ± 0.11 |
| *PAB1-GFP esa1-ts*  37°C | 0.03 ± 0.05 | *PAB1-GFP esa1-ts*  37°C | 0.40 ± 0.11 |
| *PAB1-GFP* | 0.04 ± 0.02 | *PAB1-GFP* | 1.37 ± 0.60 |
| *PAB1-GFP eaf7Δ* | 0.02 ± 0.01 | *PAB1-GFP eaf7Δ* | 0.69 ± 0.24 |
| *PAB1-GFP eaf1Δ* | 0.02 ± 0.01 | *PAB1-GFP eaf1Δ* | 0.42 ± 0.07 |
| *PBP1-GFP* | 0.06 ± 0.01 | *PBP1-GFP* | 0.93 ± 0.24 |
| *PBP1-GFP eaf7Δ* | 0.05 ± 0.01 | *PBP1-GFP eaf7Δ* | 0.98 ± 0.12 |
| *PBP1-GFP eaf1Δ* | 0.07 ± 0.02 | *PBP1-GFP eaf1Δ* | 0.74 ± 0.15 |
| *PUB1-GFP* | 0.05 ± 0.02 | *PUB1-GFP* | 3.33 ± 0.76 |
| *PUB1-GFP eaf7Δ* | 0.06 ± 0.02 | *PUB1-GFP eaf7Δ* | 2.86 ± 1.65 |
| *PUB1-GFP eaf1Δ* | 0.05 ± 0.03 | *PUB1-GFP eaf1Δ* | 0.45 ± 0.31 |
| *PAB1-GFP gcn5Δ* | 0.04 ± 0.03 | *PAB1-GFP gcn5Δ* | 1.01 ± 0.02 |
| *PAB1-GFP eaf7Δ gcn5Δ* | 0.03 ± 0.01 | *PAB1-GFP eaf7Δ gcn5Δ* | 0.65 ± 0.03 |
| *snf1Δ* *[pPAB1-GFP]* | 0.04 ± 0.01 | *snf1Δ* *[pPAB1-GFP]* | 1.22 ± 0.11 |
| *hxk2Δ* *[pPAB1-GFP]* | 0.04 ± 0.02 | *hxk2Δ* *[pPAB1-GFP]* | 0.98 ± 0.17 |
|  |  | *PAB1-GFP*  + acetate | 0.13 ± 0.01 |
| *PAB1-GFP icl1Δ* | 0.03 ± 0.01 | *PAB1-GFP icl1Δ* | 1.06 ± 0.11 |
|  |  | *PAB1-GFP icl1Δ*  + acetate | 0.14 ± 0.04 |
|  |  | *PAB1-GFP eaf7Δ*  + acetate | 0.07 ± 0.03 |
|  |  | *PAB1-GFP eaf7Δ gcn5Δ*  + acetate | 0.03 ± 0.01 |
| *PAB1-GFP acs1Δ* | 0.06 ± 0.02 | *PAB1-GFP acs1Δ* | 1.21 ± 0.02 |
| *PAB1-GFP eaf7Δ acs1Δ* | 0.01 ± 0.01 | *PAB1-GFP eaf7Δ acs1Δ* | 0.73 ± 0.10 |
| *tet07-ACC1 [pPAB1-GFP]*  - doxycycline | 0.03 ± 0.02 | *tet07-ACC1 [pPAB1-GFP]*  - doxycycline | 0.82 ± 0.08 |
| *tet07-ACC1 [pPAB1-GFP]*  + doxycycline | 0.05 ± 0.03 | *tet07-ACC1 [pPAB1-GFP]*  + doxycycline | 0.44 ± 0.05 |
| *tet07-ACC1 eaf7Δ [pPAB1-GFP]*  - doxycycline | 0.07 ± 0.03 | *tet07-ACC1 eaf7Δ [pPAB1-GFP]*  - doxycycline | 0.36 ± 0.14 |
| *tet07-ACC1 eaf7Δ [pPAB1-GFP]*  + doxycycline | 0.05 ± 0.01 | *tet07-ACC1 eaf7Δ [pPAB1-GFP]*  + doxycycline | 0.26 ± 0.01 |
|  |  | *PAB1-GFP*  + galactose | 1.48 ± 0.17 |
|  |  | *PAB1-GFP*  + ethanol | 0.73 ± 0.12 |
|  |  | *PAB1-GFP*  + citrate | 1.58 ± 0.32 |
|  |  | *PAB1-GFP*  + pyruvate | 2.21 ± 0.36 |
| *tet07-ACS2 PAB1-GFP*  - doxycycline | 0.03 ± 0.02 | *tet07-ACS2 PAB1-GFP*  - doxycycline | 1.81 ± 0.29 |
| *tet07-ACS2 PAB1-GFP*  + doxycycline | 0.05 ± 0.04 | *tet07-ACS2 PAB1-GFP*  + doxycycline | 2.31 ± 0.31 |
| *PAB1-GFP*  33°C | 0.04 ± 0.02 | *PAB1-GFP*  33°C | 1.44 ± 0.14 |
| *PAB1-GFP*  37°C | 0.07 ± 0.01 | *PAB1-GFP*  37°C | 0.20 ± 0.04 |
| *PAB1-GFP acs2-ts*  25°C | 0.04 ± 0.05 | *PAB1-GFP acs2-ts*  25°C | 1.29 ± 0.25 |
| *PAB1-GFP acs2-ts*  33°C | 0.01 ± 0.02 | *PAB1-GFP acs2-ts*  33°C | 1.45 ± 0.19 |
| *PAB1-GFP acs2-ts*  37°C | 0.02 ± 0.02 | *PAB1-GFP acs2-ts*  37°C | 0.08 ± 0.11 |
| *PAB1-GFP acs2-ts acs1Δ*  25°C | 0.06 ± 0.05 | *PAB1-GFP acs2-ts acs1Δ*  25°C | 0.87 ± 0.16 |
| *PAB1-GFP acs2-ts acs1Δ*  33°C | 0.02 ± 0.03 | *PAB1-GFP acs2-ts acs1Δ*  33°C | 1.51 ± 0.23 |
| *PAB1-GFP acs2-ts acs1Δ*  37°C | 0.07 ± 0.04 | *PAB1-GFP acs2-ts acs1Δ*  37°C | 0.11 ± 0.09 |
| + glucose LSM1 FOCI (P-Bodies) | | - glucose LSM1 FOCI (P-Bodies) | |
| *LSM1-GFP* | 0.22 ± 0.07 | *LSM1-GFP* | 3.63 ± 0.22 |
| *LSM1-GFP eaf7Δ* | 0.12 ± 0.03 | *LSM1-GFP eaf7Δ* | 2.87 ± 0.17 |
| *LSM1-GFP eaf1Δ* | 0.15 ± 0.10 | *LSM1-GFP eaf1Δ* | 3.13 ± 1.76 |
|  |  | *LSM1-GFP*  +acetate | 5.14 ± 0.80 |

A least three independent biological replicates for each yeast strain and condition were performed with 50 cells/replicate scored for the number of SGs/cell. From these values, the average foci per cell was determined; +/- standard error of the mean.
