# Supplementary material for: Lysine acetyltransferase NuA4 and acetyl-CoA regulate glucose-deprived stress granule formation in Saccharomyces cerevisiae
Source: PLoS Genet. 2017 Feb 23;13(2):e1006626. doi: 10.1371/journal.pgen.1006626 (PMC5344529; doi:10.1371/journal.pgen.1006626)
Supplement: S2 Table — Each mutant listed was transformed with PAB1-GFP::URA::CEN plasmid (pBK 192), cultured in SCD-URA medium (+glucose) at 30°C and exponential-phase cells were subjected to 10 minutes of glucose deprivation (-glucose) and immediately accessed for Pab1-GFP foci (SGs). Results are the average of the three biological replicates, a minimum of 100 cells per replicate were scored. Statistical significance at a p-Value < 0.05 was determined using an unpaired t-test. (DOCX) [file pgen.1006626.s009.docx]

**S2 Table**. **Systematic assessment of KAT and KDAC mutants for a role in glucose-deprived stress granule formation**.

|  | Strains | + glucose | | - glucose | |
| --- | --- | --- | --- | --- | --- |
|  |  | % of cells with SGs | Unpaired t-test  (p value) | % of cells with SGs | Unpaired t-test  (p value) |
|  | WT | 10.2 ± 0.8 |  | 69.7 ± 7.1 |  |
|  | *pbp1Δ* | 13.0 ± 3.3 | 0.4584 | 32.5 ± 5.2 | 0.0134 |
|  | *pub1Δ* | 17.9 ± 6.0 | 0.2733 | 40.6 ± 2.1 | 0.0170 |
|  | *eaf1Δ* | 17.4 ± 2.0 | 0.0304 | 32.1 ± 7.8 | 0.0233 |
| NuA4 | *eaf3Δ* | 10.1 ± 2.3 | 0.9061 | 26.2 ± 2.2 | 0.0074 |
|  | *eaf5Δ* | 11.6 ± 0.8 | 0.4327 | 28.0 ± 5.0 | 0.0045 |
|  | *eaf7 Δ* | 10.8 ± 1.4 | 0.8206 | 25.0 ± 1.4 | 0.0038 |
|  | *gcn5Δ* | 21.5 ± 6.7 | 0.1701 | 45.2 ± 4.4 | 0.0425 |
|  | *rtt109Δ* | 16.2 ± 3.8 | 0.1919 | 60.9 ± 6.4 | 0.4106 |
| KATs | *elp3Δ* | 16.1 ± 2.8 | 0.1172 | 57.1 ± 2.6 | 0.1711 |
|  | *hat1Δ* | 17.5 ± 3.6 | 0.1134 | 58.8 ± 7.1 | 0.3385 |
|  | *sas2Δ* | 17.3 ± 5.0 | 0.2363 | 60.3 ± 2.8 | 0.2835 |
|  | *sas3Δ* | 14.7 ± 3.1 | 0.2311 | 56.6 ± 6.3 | 0.2409 |
|  | *hpa2Δ* | 16.9 ± 4.1 | 0.1836 | 60.4 ± 8.7 | 0.4521 |
| Putative KATs | *hpa3Δ* | 15.5 ± 2.7 | 0.1289 | 72.1 ± 5.7 | 0.8023 |
|  | *spt10Δ* | 20.7 ± 7.8 | 0.2543 | 66.2 ± 10.6 | 0.7973 |
|  | *rpd3Δ* | 26.5 ± 7.6 | 0.0989 | 63.9 ± 6.9 | 0.5908 |
|  | *hos1Δ* | 16.3 ± 2.4 | 0.0762 | 64.1 ± 5.0 | 0.5539 |
|  | *hos2Δ* | 12.7 ± 0.8 | 0.0901 | 67.7 ± 3.1 | 0.8056 |
|  | *hos3Δ* | 24.7 ± 3.2 | 0.0121 | 60.1 ± 7.5 | 0.4070 |
| KDACs | *sir2Δ* | 17.6 ± 4.2 | 0.1575 | 65.1 ± 3.7 | 0.5592 |
|  | *hst1Δ* | 16.9 ± 2.7 | 0.0761 | 58.8 ± 3.6 | 0.2398 |
|  | *hst2Δ* | 18.0 ± 5.1 | 0.2052 | 66.1 ± 2.6 | 0.6601 |
|  | *hst3Δ* | 17.3 ± 5.0 | 0.2333 | 61.7 ± 3.6 | 0.3685 |
|  | *hst4Δ* | 18.4 ± 3.0 | 0.6016 | 65.2 ± 6.9 | 0.6735 |
|  | *hda1Δ* | 13.8 ± 4.0 | 0.4248 | 58.6 ± 0.7 | 0.1930 |

Each mutant listed was transformed with *PAB1-GFP::URA::CEN* plasmid (pBK 192), cultured in SCD-URA medium (+glucose) at 30^o^C and exponential-phase cells were subjected to 10 minutes of glucose deprivation (-glucose) and immediately accessed for Pab1-GFP foci (SGs). Results are the average of the three biological replicates, a minimum of 100 cells per replicate were scored. Statistical significance at a p-Value < 0.05 was determined using an unpaired t-test.
