# Supplementary material for: Lysine acetyltransferase NuA4 and acetyl-CoA regulate glucose-deprived stress granule formation in Saccharomyces cerevisiae
Source: PLoS Genet. 2017 Feb 23;13(2):e1006626. doi: 10.1371/journal.pgen.1006626 (PMC5344529; doi:10.1371/journal.pgen.1006626)
Supplement: S3 Table — (DOCX) [file pgen.1006626.s010.docx]

**S3 Table: Strains and plasmid used in this study.**

| Strains | Genotype | Reference |
| --- | --- | --- |
| YKB3263 | *MATa leu2Δ0 ura3Δ0 his3Δ1 met15Δ0 [pPAB1-GFP::URA]* | This study |
| YKB3262 | *MATa leu2Δ0 ura3Δ0 his3Δ1 met15Δ0 pbp1Δ::KANMX [pPAB1-GFP::URA]* | This study |
| YKB3261 | *MATa leu2Δ0 ura3Δ0 his3Δ1 met15Δ0 pub1Δ::KANMX [pPAB1-GFP::URA]* | This study |
| YKB3260 | *MATa leu2Δ0 ura3Δ0 his3Δ1 met15Δ0 eaf1Δ::NATMX [pPAB1-GFP::URA]* | This study |
| YKB3729 | *MATa leu2Δ0 ura3Δ0 his3Δ1 met15Δ0 eaf7Δ::KANMX [pPAB1-GFP::URA]* | This study |
| YKB3114 | *MATa leu2Δ0 ura3Δ0 his3Δ1 met15Δ0 PAB1-GFP::HIS* | GFP collection |
| YKB3855 | *MATa leu2Δ lys2Δ0 esa1ΔHIS3 esa1-L245P::URA3 PAB1-GFP::HIS* | This study |
| YKB3382 | *MATa leu2Δ0 ura3Δ0 his3Δ1 met15Δ0 PAB1-GFP::HIS eaf1Δ::NATMX* | This study |
| YKB3336 | *MATa leu2Δ0 ura3Δ0 his3Δ1 met15Δ0 PAB1-GFP::HIS eaf7Δ::KANMX* | This study |
| YKB3115 | *MATa leu2Δ0 ura3Δ0 his3Δ1 met15Δ0 PUB1-GFP::HIS* | GFP collection |
| YKB3337 | *MATa leu2Δ0 ura3Δ0 his3Δ1 met15Δ0 PUB1-GFP::HIS eaf7Δ::KANMX* | This study |
| YKB3339 | *MATa leu2Δ0 ura3Δ0 his3Δ1 met15Δ0 PUB1-GFP::HIS eaf1Δ::NATMX* | This study |
| YKB3258 | *MATa leu2Δ0 ura3Δ0 his3Δ1 met15Δ0 PBP1-GFP::HIS* | GFP collection |
| YKB3335 | *MATa leu2Δ0 ura3Δ0 his3Δ1 met15Δ0 PBP1-GFP::HIS eaf7Δ::KANMX* | This study |
| YKB3338 | *MATa leu2Δ0 ura3Δ0 his3Δ1 met15Δ0 PBP1-GFP::HIS eaf1Δ::NATMX* | This study |
| YKB3710 | *MATa leu2Δ0 ura3Δ0 his3Δ1 met15Δ0 LSM1-GFP::HIS* | GFP collection |
| YKB3718 | *MATa leu2Δ0 ura3Δ0 his3Δ1 met15Δ0 LSM1-GFP::HIS eaf7Δ::KANMX* | This study |
| YKB3717 | *MATa leu2Δ0 ura3Δ0 his3Δ1 met15Δ0 LSM1-GFP::HIS eaf1Δ::NATMX* | This study |
| YKB4116 | *MATa leu2Δ0 ura3Δ0 his3Δ1 met15Δ0 PAB1-GFP::HIS gcn5Δ::KANMX* | This study |
| YKB4118 | *MATa leu2Δ0 ura3Δ0 his3Δ1 met15Δ0 PAB1-GFP::HIS gcn5Δ::KANMX* *eaf7Δ::KANMX* | This study |
| BY4741  (YKB 1079) | *MATa leu2Δ0 ura3Δ0 his3Δ1 met15Δ0* | DMA collection |
| YKB3333 | *MATa leu2Δ0 ura3Δ0 his3Δ1 met15Δ0 eaf1Δ::NATMX* | This study |
| YKB3292 | *MATa leu2Δ0 ura3Δ0 his3Δ1 met15Δ0 eaf7Δ::KANMX* | DMA collection |
| YKB4030 | *MATa leu2Δ0 ura3Δ0 his3Δ1 met15Δ0 [pPAB1-GFP::URA]* | This study |
| YKB4342 | *MATa leu2Δ0 ura3Δ0 his3Δ1 met15Δ0 snf1Δ::KANMX [pPAB1-GFP::URA]* | This study |
| YKB4343 | *MATa leu2Δ0 ura3Δ0 his3Δ1 met15Δ0 hxk2Δ::KANMX [pPAB1-GFP::URA]* | This study |
| YKB4010 | *MATa leu2Δ0 ura3Δ0 his3Δ1 met15Δ0 PAB1-GFP::HIS icl1Δ::KANMX* | This study |
| YKB4047 | *MATa leu2Δ1::tTA-LEU2 trp1-63 ura3-52 tet07-ACC1* | (56) |
| YKB4048 | *MATa leu2Δ1::tTA-LEU2 trp1-63 ura3-52 tet07-ACC1 [pPAB1-GFP::URA]* | This study |
| YKB4132 | *MATa leu2Δ0 ura3Δ0 his3Δ1 met15Δ0 PAB1-GFP::HIS acs1Δ::KANMX* | This study |
| YKB4287 | *MATa leu2Δ0 ura3Δ0 his3Δ1 met15Δ0 PAB1-GFP::HIS acs1Δ::KANMX eaf7Δ::KANMX* | This study |
| YKB4122 | *MATa leu2Δ0 ura3Δ0 his3Δ1 met15Δ0 PAB1-GFP::KANMX acs2Δ::HYGMX [pHT215, acs2-Ts1-CEN-URA3]* | This study |
| YKB4121 | *MATa leu2Δ0 ura3Δ0 his3Δ1 met15Δ0 PAB1-GFP::KANMX acs1Δ::HIS3 acs2Δ::HYGMX [pHT215, acs2-Ts1-CEN-URA3]* | This study |
| YKB4227 | *MATa leu2Δ0 ura3Δ0 his3Δ1 met15Δ0 PAB1-GFP::HIS ach1Δ::KANMX* | This study |
| YKB4228 | *MATa leu2Δ0 ura3Δ0 his3Δ1 met15Δ0 PAB1-GFP::HIS mpc15Δ::KANMX* | This study |
| YKB4249 | *MATa leu2Δ1::tTA-LEU2 trp1-63 ura3-52 tet07-ACC1 eaf7Δ::NATMX [pPAB1-GFP::URA]* | This study |
| YKB4246 | *URA3::CMV-tTA MATa his3-1 leu2-0 met15-0 kanR-tetO7-ACS2 PAB1-GFP::HIS* | This study |
| YKB3954 | *MATa leu2Δ0 ura3Δ0 his3Δ1 met15Δ0 ACC1-GFP::HIS* | GFP collection |
| YKB3929 | *MATa leu2Δ0 ura3Δ0 his3Δ1 met15Δ0 ACC1-GFP::HIS eaf1Δ::KANMX* | This study |
| YKB3930 | *MATa leu2Δ0 ura3Δ0 his3Δ1 met15Δ0 ACC1-GFP::HIS eaf7Δ::KANMX* | This study |
| Plasmid | **Details** | **Reference** |
| pRP1362  (pKB192) | Pab1-GFP; Cen; *URA3* marker; *[pPAB1-GPF::URA]* | (17) |
